# Structural basis of the interaction between Topoisomerase IIIβ and the TDRD3 auxiliary factor

**DOI:** 10.1038/srep42123

**Published:** 2017-02-08

**Authors:** Sakurako Goto-Ito, Atsushi Yamagata, Tomio S. Takahashi, Yusuke Sato, Shuya Fukai

**Affiliations:** 1 Structural Biology Laboratory, Structural Life Science Division, Synchrotron Radiation Research Organization and Institute of Molecular and Cellular Biosciences, The University of Tokyo, Tokyo 113-0032, Japan; 2 CREST, JST, Saitama 332-0012, Japan; 3 Department of Computational Biology and Medical Sciences, Graduate School of Frontier Sciences, The University of Tokyo, Chiba 277-8501, Japan

## Abstract

Topoisomerase IIIβ (TOP3β) is a DNA/RNA topoisomerase that has been implicated in epigenetic or translational control of gene expression. In cells, TOP3β co-exists with its specific auxiliary factor, TDRD3. TDRD3 serves as a scaffold protein to recruit TOP3β to its DNA/RNA substrates accumulating in specific cellular sites such as methylated chromatins or neural stress granules. Here we report the crystal structures of the catalytic domain of TOP3β, the DUF1767–OB-fold domains of TDRD3 and their complex at 3.44 Å, 1.62 Å and 3.6 Å resolutions, respectively. The toroidal-shaped catalytic domain of TOP3β binds the OB-fold domain of TDRD3. The TDRD3 OB-fold domain harbors the insertion loop, which is protruding from the core structure. Both the insertion loop and core region interact with TOP3β. Our pull-down binding assays showed that hydrophobic characters of the core surface and the amino- and carboxy-terminal regions of the insertion loop are essential for the interaction. Furthermore, by comparison with the structure of the homologous Topoisomerase IIIα (TOP3α)–RMI1 complex, we identified Arg96, Val109, Phe139 and the short insertion loop of TDRD3 as the critical structural elements for the specific interaction with TOP3β to avoid the non-cognate interaction with TOP3α.

Expression or inheritance of genetic information (*i.e.*, transcription, replication or recombination) is associated with unwinding of DNA duplexes, which causes their topological distortions that impede these genetic processes. DNA topoisomerases play critical roles in resolving the topological problems by temporal cleavage, strand passage and resealing of the DNA^1^,^2^. During these reactions, topoisomerases transiently trap the end of the cleaved DNA by covalent bonding via catalytic tyrosine residues. Topoisomerases that can cleave only one strand of the DNA are defined as type I, whereas those that can introduce a double-strand break as type II^1^,^2^. The type II enzyme functions mainly in segregation of the catenated DNA after replication, where a double strand is passed over through a break in another double strand. At least one type II DNA topoisomerase is required for all organisms examined. The type I enzyme is further classified into two structurally distinct subtypes, IA and IB. The type IA enzyme covalently bonds to the 5′-end of the cleaved DNA, whereas the type IB enzyme bonds to the 3′-end. The type IB enzyme, which has been found in eukaryotes, animal viruses, mammalian mitochondria and some bacteria and archaea^3^,^4^,^5^,^6^,^7^, relaxes the supercoiling stress formed during transcription and replication. On the other hand, the type IA enzyme has been found in all organisms and play divergent roles, including resolution of recombination intermediates and maintenance of DNA supercoiling. All organisms possess multiple topoisomerases, corresponding to various types of topological problems.

Topoisomerase III (TOP3) is a member of the type IA topoisomerase family, which functions mainly in relaxing the supercoiled DNA and in resolving recombination intermediates during meiosis and DNA repair. Most bacteria and fungi have one TOP3 enzyme, whereas metazoans and several fungi have two TOP3 enzymes, TOP3α and β^1^,^2^,^8^. Bacterial or fungal TOP3 is equivalent to TOP3α. Eukaryotic TOP3α and β are unique in that they interact with their specific auxiliary factors, RMI1 and TDRD3, respectively, and coexist with them in the cell^9^,^10^,^11^,^12^,^13^. RMI1 can bridge between the TOP3α–BLM helicase complex and RMI2 to assemble into a higher molecular-weight complex called a BLM dissolvasome, which is also conserved in yeast (Sgs1–Top3–Rmi1 complex)^14^,^15^. The BLM dissolvasome is indispensable for DNA repair pathways following the DNA double strand break and for formation and resolution of recombination intermediates^16^,^17^,^18^,^19^. Therefore, TOP3α is an essential DNA topoisomerase^20^,^21^.

TOP3β is an atypical topoisomerase that can recognize both DNA and RNA as the substrates^12^,^13^,^22^,^23^,^24^,^25^,^26^. Although TOP3β is not essential for viability, TOP3β-null mice are deficient in fertility and immunity^27^,^28^,^29^. Furthermore, loss of TOP3β causes schizophrenia and cognitive impairment, along with abnormal synapse formation^12^,^13^. In neurons, TOP3β and TDRD3 form a ternary complex with the fragile X mental retardation protein (FMRP) through the interaction between the TDRD3 C-terminal domain and FMRP (known as a TTF complex)^12^,^13^,^30^. FMRP binds primarily to the coding region of more than 840 mRNAs that have neural functions^31^. The RNA-binding-deficient single missense mutation in FMRP leads directly to fragile X syndrome, showing the importance of RNA regulation by FMRP. As a component of the TTF complex, TOP3β regulates the expression of mRNAs that are important in neurodevelopment^12^. TOP3β is required for the proper translational regulation by FMRP in neurons.

The TOP3β–TDRD3 complex also functions in epigenetic regulation^32^. The Tudor domain of TDRD3 recognizes the asymmetric di-methylation (me2a) of histone H4 Arg3 and H3 Arg17 modifications, which activate transcription^33^. The Tudor domain also recognizes the Arg1810 me2a modification in the CTD of RNA polymerase II^34^. These interactions recruit the TOP3β–TDRD3 complex to the actively transcribed genes. During transcription by RNA polymerase II, the newly transcribed RNA can anneal back to the template DNA and forms stable DNA/RNA hybrid strands called R loops. TOP3β resolves the R loops at the heavily transcribed genes to prevent the R loop accumulation and promote transcription^32^.

Despite the functional importance of TOP3β, no structural information of TOP3β and the TOP3β-interacting domains of TDRD3 has been available. Accordingly, it remains unclear how TDRD3 regulates the TOP3β activity. In this study, we solved the crystal structures of TOP3β, TDRD3 and the TOP3β–TDRD3 complex. These structures reveal the atomic details of the TOP3β–TDRD3 interaction. Further biochemical studies showed the critical structural elements of the interaction. Comparison with the TOP3α–RMI1 complex elucidated the mechanism for selection of the specific auxiliary factor.

## Results

### Overall structures

**Figure 1:**
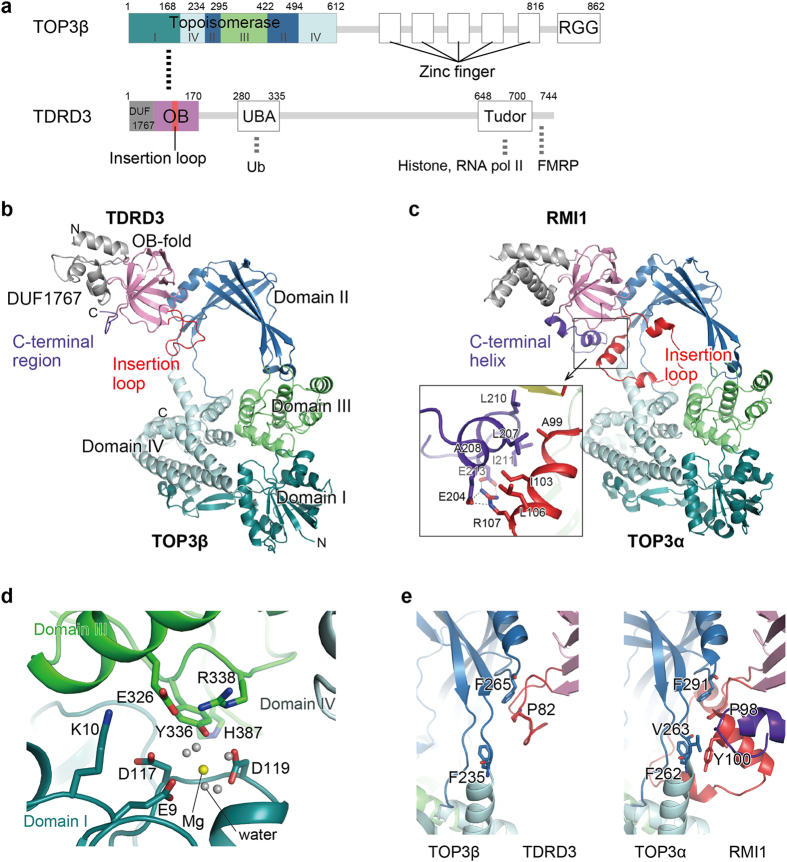
Structures of TOP3β, TDRD3 and the TOP3β–TDRD3 complex. (**a**) Domain organizations of TOP3β and TDRD3. Thick dashed lines represent molecular interactions. (**b**) Overall structure of the TOP3β–TDRD3 complex. (**c**) Overall structure of the TOP3α–RMI1 complex. The close-up view shows the interaction between the C-terminal helix of TOP3α and the insertion loop of RMI1. (**d**) Catalytic pocket of apo TOP3β. The bound magnesium ion and its coordinated water molecules are shown as yellow and grey spheres, respectively. (**e**) Expected pivot points of TOP3β and TOP3α in the TOP3β–TDRD3 and TOP3α–RMI1 complexes, respectively. The coloring scheme is the same as that of (**b,c**).

TOP3β consists of a topoisomerase domain (TOPO domain), five zinc-finger domains (ZF domains) and an RGG domain (Fig. 1a). TDRD3 contains a long, unstructured region in the middle, which connects the N-terminal DUF1767, OB-fold and ubiquitin-associated (UBA) domains and the C-terminal Tudor domain. We determined the crystal structures of human TOP3β TOPO domain, TDRD3 DUF1767 and OB domains (DUF–OB) and their complex at 3.44 Å, 1.62 Å and 3.6 Å resolutions, respectively (Fig. 1b and Supplementary Fig. 1a–c). Overall, the TOP3β–TDRD3 structure is similar to the TOP3α–RMI1 structure^35^ (Fig. 1b,c and Supplementary Fig. 1b,d). The TOPO domain of TOP3β exhibits a toroidal-shaped structure, which is characteristic of the type IA topoisomerase. The TOPO domain is further divided into four subdomains (domains I–IV) (Fig. 1a,b)^36^. The cavity formed by domains I, III and IV constitutes a Mg^2+^-bound catalytic pocket (Fig. 1d), where the catalytic residues are spatially arranged in a manner similar to those of other type IA topoisomerases (Fig. 1d and Supplementary Fig. 2)^35^,^37^,^38^. Previously reported *E. coli* TOP3 structures showed a transition from a closed conformation to an open conformation in the type IA enzyme upon binding of the substrate DNA^39^ (Supplementary Fig. 2d,e). Our substrate-free TOP3β structures adopted the closed conformation (Supplementary Fig. 2a). Binding of TDRD3 did not affect the spatial arrangement of domains I, III and IV (Supplementary Fig. 2b). In the type IA topoisomerase, one strand of the substrate DNA is cleaved and trapped in the catalytic pocket by covalent bonding between its 5′-end and the catalytic tyrosine residue, which enables the opposed strand to pass over the cleaved strand. For this strand passage reaction, domain III has been proposed to detach from domain I with a swing motion of domains II and IV^36^. In this context, the tight stacking interactions of TOP3α Phe262, Val263 and Phe291 with RMI1 Pro98 and Tyr100 in the TOP3α–RMI1 complex are expected to serve as the pivot point for the dynamic motion of domains II–IV^35^ (Fig. 1e) by structural analogy to *E. coli* TOP1. Although the two phenylalanine residues at this pivot point are conserved in TOP3β, the stacking interaction is formed only between TOP3β Phe265 and TDRD3 Pro82. Binding of TDRD3 might have less effect on the motion of domains II–IV than that of RMI1.

**Figure 2:**
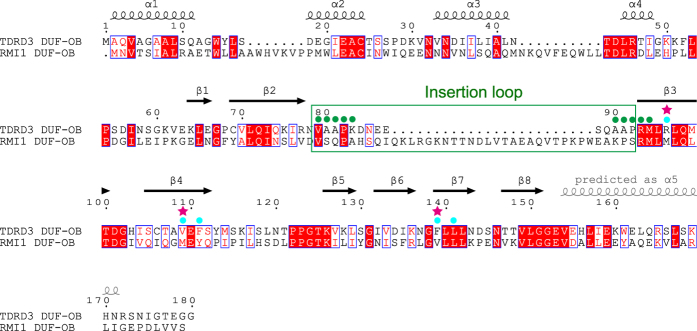
Amino-acid sequence alignment of TDRD3 and RMI1. The TOP3β-interacting residues in the insertion loop and the core region are marked with green and cyan dots, respectively. The residues critical for preferential binding to TOP3β are marked with magenta stars.

The N-terminal DUF1767 domain of TDRD3 is composed of four α-helices, followed by the β-barrel structure of the OB-fold domain (Fig. 1b and Supplementary Fig. 1b). The DUF1767 structures of TDRD3 and RMI1 are topologically similar but differ in size and orientation relative to the OB-fold domain (Supplementary Fig. 1b,d). The DUF1767 domain is missing in many species on *Ensembl* database^40^, suggesting that the DUF1767 domain is dispensable for the function. Our binding analysis using the size-exclusion column chromatography showed that the TDRD3 OB-fold domain alone could bind TOP3β (Supplementary Fig. 3a). The OB-fold domain of TDRD3 contains an insertion loop between β2 and β3 (Val79–Pro92), which is 23 residues shorter than that of RMI1 (Val95–Ser132) (Figs. 1b,c and 2 and Supplementary Fig. 1b,d). In the TOP3α–RMI1 structure, the insertion loop of RMI1 interacts with the C-terminal α-helix of the RMI1 OB-fold domain (Fig. 1c), whereas a similar interaction is not observed in TDRD3 (Fig. 1b). Concomitantly, the C-terminal region of the TDRD3 OB-fold domain is not folded into an α-helix, contrary to the secondary structure prediction by PSIPRED (Fig. 2). Limited digestion with chymotrypsin cleaved the C-terminus of TDRD3 OB-fold at Trp161, which is positioned in the middle of the predicted α-helix (Fig. 2 and Supplementary Fig. 3b). This result suggests that the C-terminal region of the TDRD3 OB-fold domain is unfolded in solution, consistent with the present structure.

The insertion loop of TDRD3 is completely disordered in the apo TDRD3 and becomes ordered upon binding to TOP3β (Fig. 1b and Supplementary Fig. 1b) with some flexibility, as judged from high temperature factors (Supplementary Fig. 4a). Comparison among the two apo TDRD3 structures in the asymmetric unit and the TOP3β-bound TDRD3 structure exhibited two different conformations around the N- and C-terminal ends of β3 and β4, respectively (Supplementary Fig. 4b–d). β3 and β4 in one of the two apo TDRD3 molecules and the TOP3β-bound TDRD3 are one residue shorter than those in the other apo TDRD3 molecule. The Arg93-mediated hydrogen bonds stabilize the shorter β3 and β4, which might be appropriate for binding to TOP3β (Supplementary Fig. 4c,d).

### TOP3β–TDRD3 interaction

The interaction between TOP3β and TDRD3 occurs between domain II of TOP3β and the OB-fold domain of TDRD3, consistent with our finding that the TDRD3 OB-fold domain is sufficient for binding to TOP3β (Fig. 1b and Supplementary Fig. 3a). Domain II of TOP3β appears more flexible than domains I, III and IV in the present TOP3β structures (one from the complex and two from the apo TOP3β in the asymmetric unit), indicative of its intrinsic flexibility for the induced fit binding to TDRD3 (Supplementary Fig. 4e).

**Figure 3:**
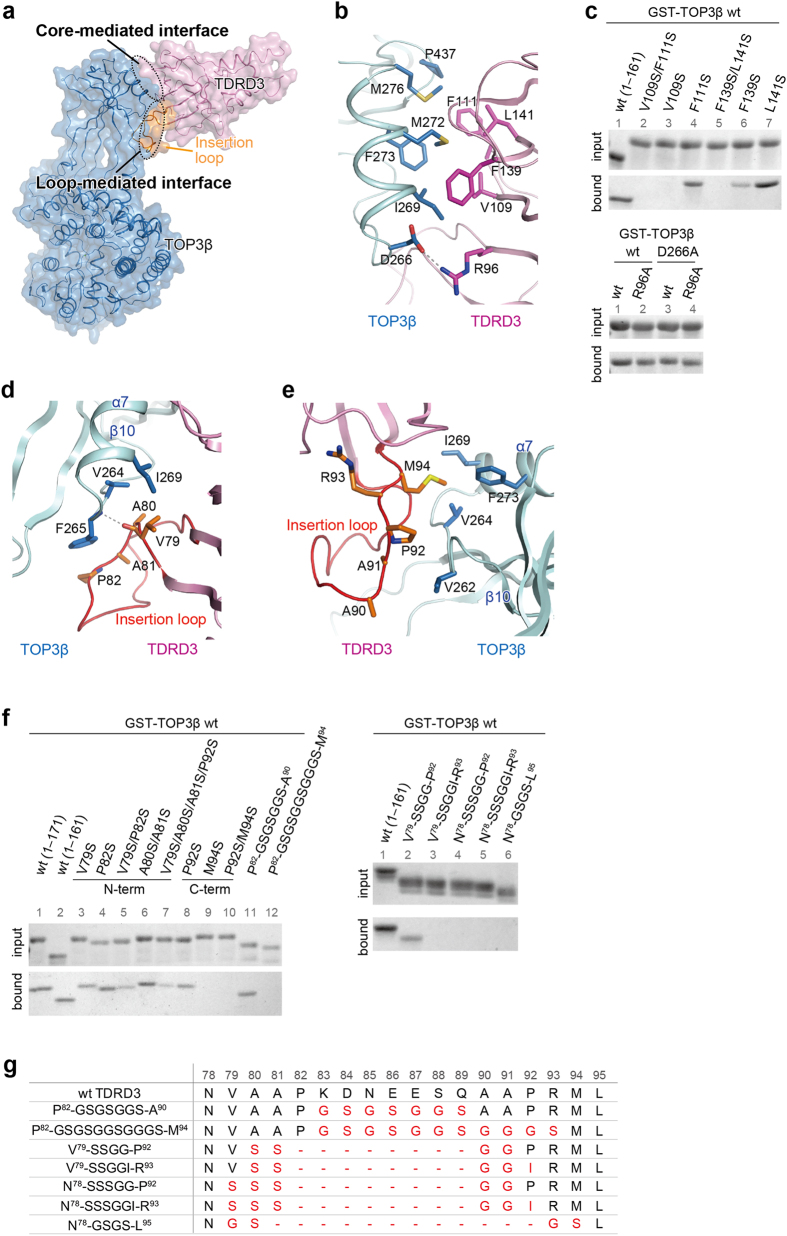
Interactions between TOP3β and TDRD3. (**a**) Surface representation of the TOP3β–TDRD3 complex. TDRD3 is colored in pink, except that the insertion loop is colored in orange. TOP3β is colored in blue. The core- and loop-mediated interfaces are encircled by dotted lines. (**b**) The core-mediated interactions between TOP3β and TDRD3. (**c**) GST pull-down assays of TDRD3 mutants to assess the core-mediated interactions. The GST-TOP3β-bound resins were incubated with 0.5 μM TDRD3 wild-type or mutant proteins. Shown are the cropped gel images. The contrast was adjusted for clarity. Quantitative analyses and original full-length gel images corresponding to the top and bottom panels are shown in Supplementary Fig. 5a,b, respectively. (**d**) Interactions between TOP3β and the N-terminal region of the TDRD3 insertion loop. (**e**) Interactions between TOP3β and the C-terminal region of the TDRD3 insertion loop. (**f**) GST pull-down assays of TDRD3 mutants to assess the loop-mediated interactions. The GST-TOP3β-bound resins were incubated with 0.2 μM or 1.0 μM TDRD3 proteins shown in left or right panels, respectively. Shown are the cropped gel images. The contrast was adjusted for clarity. Quantitative analyses and original full-length gel images corresponding to the left and right panels are shown in Supplementary Fig. 5c,d, respectively. (**g**) Amino-acid sequences of the insertion loop in the TDRD3 mutants shown in (**f**). The residue numbers of wild-type TDRD3 are shown in the top. The mutated residues are colored in red.

Both the core region and insertion loop of the TDRD3 OB-fold domain bind domain II of TOP3β with buried surface areas of 472 Å^2^ and 367 Å^2^, respectively (Fig. 3a). The core-mediated interaction is primarily hydrophobic: Val109, Phe111, Phe139 and Leu141 of TDRD3 and Ile269, Met272, Phe273, Met276 and Pro437 of TOP3β engage in this hydrophobic interaction (Fig. 3b). In addition, a hydrogen bond is formed between TDRD3 Arg96 and TOP3β Asp266. To assess the contribution of these residues to the TDRD3–TOP3β interaction, we tested binding of TOP3β or TDRD3 mutants by GST pull-down assays. Quantification of the affinities by the surface plasmon resonance analysis was unsuccessful due to technical difficulty. Instead, the bound proteins were quantified by densitometry to allow easy comparison between wild type and mutants or between different mutants. The hydrophobic residues of TDRD3 involved in the core-mediated interaction were mutated to serine residues to abrogate the hydrophobicity. As shown in Fig. 3c and Supplementary Fig. 5a, the V109S single mutant or the F139S/L141S double mutant of TDRD3 eliminated binding to TOP3β. In contrast, the R96A mutant of TDRD3 and the D266A mutant of TOP3β showed the binding activity comparable to wild type (Fig. 3c and Supplementary Fig. 5b). Therefore, the hydrophobic character of the TDRD3 OB-fold surface is essential for the core-mediated interaction.

The insertion loop of TDRD3 interacts with the loop connecting β10 and α7 of TOP3β (Fig. 3d,e). In the N-terminal region of the insertion loop, Val79, Ala80, Ala81 and Pro82 of TDRD3 form a hydrophobic surface to interact with Val264, Phe265 and Ile269 of TOP3β (Fig. 3d). The main-chain CO group of TDRD3 Val79 forms a hydrogen bond with the main-chain NH group of TOP3β Phe265. Furthermore, in the C-terminal region of the insertion loop, Ala90, Ala91, Pro92 and Met94 of TDRD3 also form a hydrophobic surface to interact with Val262, Val264, Ile269 and Phe273 of TOP3β (Fig. 3e). We assessed the contribution of these residues to the TDRD3–TOP3β interaction by GST pull-down assays (Fig. 3f and Supplementary Fig. 5c,d). As for the mutations in the N-terminal region of the loop, the V79S/P82S double mutation obviously decreased the binding (~30% of wild-type binding) (Fig. 3f, left panel and Supplementary Fig. 5c, lane 5), whereas the V79S or P82S mutation or the A80S/A81S double mutation had little or moderate effect on the binding (~60% or higher of wild-type binding) (Fig. 3f, left panel and Supplementary Fig. 5c, lanes 3, 4, and 6). As for the mutations in the C-terminal region of the insertion loop, the P92S mutation had moderate effect on the binding (~50% of wild-type binding) (Fig. 3f, left panel and Supplementary Fig. 5c, lane 8), whereas the M94S mutation and the P92S/M94S double mutation eliminated the binding (Fig. 3f, left panel and Supplementary Fig. 5c, lanes 9 and 10). We also assessed the contribution of the middle region of the insertion loop by substituting a 7-residue Gly-Ser linker for Lys83–Gln89 (Fig. 3g). This mutant (P^82^-GSGSGGS-A^90^) retained the binding (~60% of wild-type binding), indicating that the amino-acid sequence of the middle region is not critical (Fig. 3f, left panel and Supplementary Fig. 5c, lane 11). On the other hand, the replacement of Lys83–Arg93 by an 11-residue Gly-Ser linker (P^82^-GSGSGGSGGGS-M^94^) eliminated the binding (Fig. 3f, left panel and Supplementary Fig. 5c, lane 12), indicating that Ala90–Arg93 contains indispensable elements for the binding. To ask whether the length of the insertion loop is critical for the binding, we shortened the insertion loop by substituting Ser-Ser-Gly-Gly for Ala80–Ala91 (V^79^-SSGG-P^92^). However, this mutant could still bind TOP3β (~40% of wild-type binding) (Fig. 3f, right panel and Supplementary Fig. 5d, lane 2). We further trimmed this shortcut insertion loop by substituting Gly-Ser-Gly-Ser for Val79–Met94 (N^78^-GSGS-L^95^) or mutated Val79 (N^78^-SSSGG-P^92^), Pro92 (V^79^-SSGGI-R^93^) or both (N^78^-SSSGGS-R^93^). These trimming and point mutations of the shortcut insertion loop eliminated binding to TOP3β (Fig. 3f, right panel and Supplementary Fig. 5d, lanes 3, 4, 5 and 6), indicating that both Val79 and Pro92 are required for binding of the short insertion loop-containing TDRD3 to TOP3β. On the other hand, the V79S or P92S mutation had little or moderate effect on binding of TDRD3 (containing the intact insertion loop) to TOP3β (Fig. 3f, left panel and Supplementary Fig. 5c, lanes 3 and 8). This is probably because the remaining hydrophobic interactions can compensate for the loss of the Val79- or Pro92-mediated hydrophobic interaction.

Taken together, these results indicate that the N- and C-terminal regions of the insertion loop, particularly Met94, play critical roles in binding of TDRD3 to TOP3β.

### Discrimination between TOP3α and β by TDRD3

**Figure 4:**
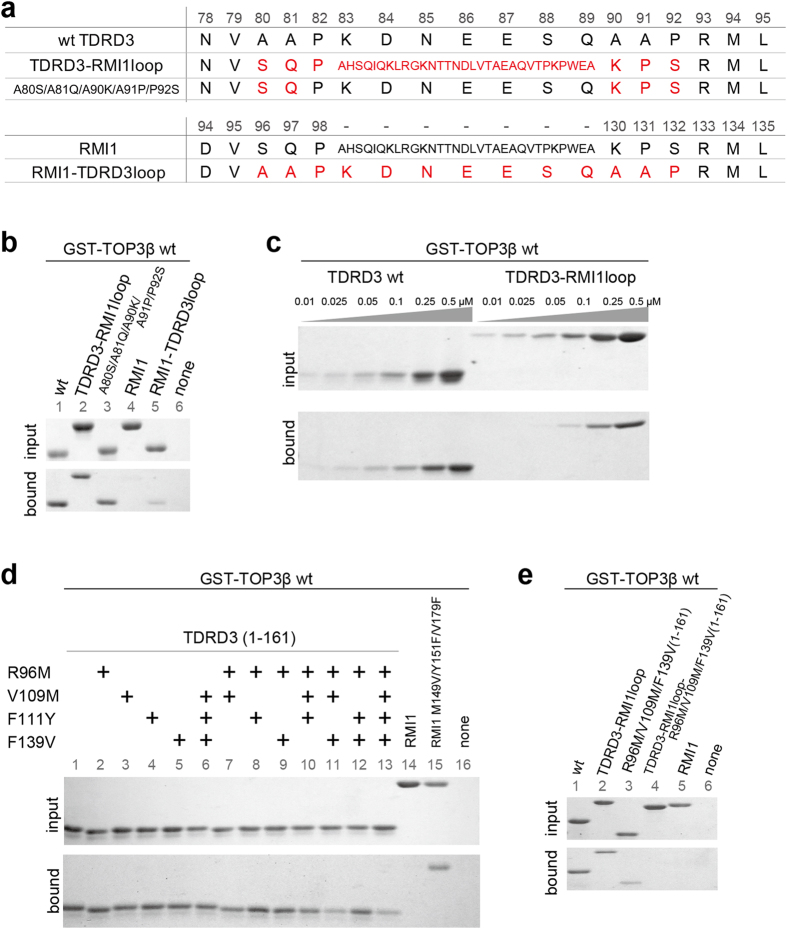
Specificity determinants for the interaction between TOP3β and TDRD3. (**a**) Amino-acid sequences of the insertion loop region in the TDRD3 mutant containing the RMI1 insertion loop (TDRD3-RMI1loop), the A80S/A81Q/A90K/A91P/P92S mutant of TDRD3 and the RMI1 mutant containing the TDRD3 insertion loop (RMI1-TDRD3loop). (**b**) GST pull-down assays of TDRD3-RMI1loop, the A80S/A81Q/A90K/A91P/P92S mutant and RMI1-TDRD3loop. The GST-TOP3β-bound resins were incubated with 1.0 μM wild-type or mutant proteins of TDRD3 or RMI1. Shown are the cropped gel images. The contrast was adjusted for clarity. Quantitative analyses and original full-length gel images corresponding to the left and right panels are shown in Supplementary Fig. 7a. (**c**) GST pull-down assays of TDRD3-RMI1oop at different concentrations (0.01–0.5 μM). TDRD3-RMI1loop could bind TOP3β, but with lower affinity than wild-type TDRD3. Shown are the cropped gel images. The contrast was adjusted for clarity. Quantitative analyses and original full-length gel images corresponding to this figure are shown in Supplementary Fig. 7b. (**d**) GST pull-down assays of the TDRD3 mutants containing R96M, V109M, F111Y, F139V or their combinations and the M149V/Y151F/V179F mutant of RMI1. The GST-TOP3β-bound resins were incubated with 0.5 μM wild-type or mutant proteins of TDRD3 or RMI1. Shown are the cropped gel images. The contrast was adjusted for clarity. Quantitative analyses and original full-length gel images corresponding to this figure are shown in Supplementary Fig. 7c. (**e**) GST pull-down assay of the TDRD3-RMI1oop mutant containing the R96M, V109M and F139V mutations. The GST-TOP3β-bound resins were incubated with 0.5 μM wild-type or mutant proteins of TDRD3 or RMI1. Shown are the cropped gel images. Quantitative analyses and original full-length gel images corresponding to the left and right panels are shown in Supplementary Fig. 7d.

Remarkable structural difference between TDRD3 and RMI1 is found in the insertion loop, which is involved in binding to TOP3β and α, respectively (Fig. 1b,c). We asked whether the insertion loop contributes to the discrimination between TOP3α and β. We first confirmed that the long insertion loop of RMI1 could not be overlapped with TOP3β when the TOP3β–TDRD3 and TOP3α–RMI1 structures were superposed (Supplementary Fig. 6). Then, the insertion loop was exchanged between TDRD3 and RMI1. The TDRD3 mutant containing the RMI1 insertion loop and the RMI1 mutant containing the TDRD3 insertion loop are referred hereafter to as TDRD3-RMI1loop and RMI1-TDRD3loop, respectively (Fig. 4a). As shown in Fig. 4b and Supplementary Fig. 7a (lanes 4 and 5), RMI1-TDRD3loop exhibited weak but increased binding to TOP3β (~30% of wild-type TDRD3 binding), as compared to RMI1 (~10% of wild-type TDRD3 binding), indicating that the TDRD3 loop could confer the ability to bind TOP3β on RMI1. On the other hand, TDRD3-RMI1loop retained the binding ability to TOP3β (Fig. 4b and Supplementary Fig. 7a, lane 2) with a lower affinity than wild type (Fig. 4c and Supplementary Fig. 7b), suggesting that the short TDRD3 insertion loop is favorable for binding to TOP3β. Additionally, the mutations in the N- and C-terminal regions of the TDRD3 insertion loop to mimic those of the RMI1 loop (*i.e.*, A80S/A81Q/A90K/A91P/P92S) showed little effect on binding to TOP3β (~80% of wild-type binding) (Fig. 4b and Supplementary Fig. 7a, lane 3), although both regions are critically important for the binding, as mentioned above. These findings suggest that the preferential binding of TDRD3 to TOP3β does not rely solely on the insertion loop-mediated interaction.

Therefore, we next asked whether the core-mediated interaction also contributes to the specificity towards TOP3β. Arg96, Val109, Phe111 and Phe139 of TDRD3, which are involved in the core-mediated interaction with TOP3β, are replaced by Met136, Met149, Tyr151 and Val179 in RMI1, respectively (Fig. 2). We mutated these TDRD3 residues to the corresponding RMI1 residues and tested binding to TOP3β. Although the single mutations (R96M, V109M, F111Y or F139V), the double mutations (R96M/V109M, R96M/F111Y or R96M/F139V), or the V109M/F111Y/F139V mutation of TDRD3 did not drastically decrease binding to TOP3β (~70% or higher of wild-type binding), the combination of R96M, V109M and F139V mutations obviously decreased the binding (~35% of wild-type binding). (Fig. 4d and Supplementary Fig. 7c). Furthermore, the replacement of Met149, Tyr151 and Val179 of RMI1 by the corresponding TDRD3 residues (Val, Phe and Phe, respectively) enabled RMI1 to bind TOP3β (~40% of wild-type TDRD3 binding) (Fig. 4d, lanes 14 and 15). These results indicate that the residues involved in the core-mediated interaction, especially Arg96, Val109 and Phe139, contribute to the preference of TDRD3 towards TOP3β. Finally, we simultaneously replaced the insertion loop and essential core residues of TDRD3 by those of RMI1 (TDRD3-RMI1loop-R96M/V109M/F139V) and tested binding to TOP3β. As shown in Fig. 4e and Supplementary Fig. 7d, this mutant eliminated the binding (lane 4) in contrast to TDRD3-RMI1loop or the R96M/V109M/F139V mutant, which showed ~70% or ~30% of wild-type binding, respectively.

Taken together, the residues on the surface of the TDRD3 OB-fold core are the important determinants in the selectivity to TOP3β. The short insertion loop also contributes to it. The preference of TDRD3 for TOP3β is established by the combination of the core- and insertion loop-mediated interactions.

## Discussion

The interacting residues of TOP3β and TDRD3 are conserved or replaced by functionally equivalent residues among 10 organisms examined (Supplementary Figs 8 and 9). One exception is Arg96 of human TDRD3, which is replaced by Gln (Gln96) in *D. melanogaster*. Interestingly, Asp266 of human TOP3β, the hydrogen-bonding partner of human TDRD3 Arg96, is replaced by Lys (Lys266) in *D. melanogaster* (Supplementary Fig. 9), suggesting that the Arg96-Asp266 hydrogen bond pair in human TOP3β–TDRD3 complex is replaced by the Gln96-Lys266 pair in *D. melanogaster* complex. The TDRD3-interacting residues of TOP3β are located in α7 and the loop connecting β10 and α7. The residues on α7 are totally different from the corresponding residues of TOP3α, suggesting that the TOP3β residues on α7 and the loop have diverged to gain selectivity for TDRD3. The R472Q mutation of TOP3β, which is located in domain II, has been found in schizophrenia patients^41^. Arg472 is conserved or replaced by Lys in vertebrates but not in insects (Supplementary Fig. 9). Interestingly, TOP3α and *D. melanogaster* TOP3β possess a glutamine residue in the corresponding position. The R472Q mutation may hardly affect the tertiary structure of TOP3β, as the side chain of Arg472 is exposed to the solvent and partly disordered. The R472Q mutation of TOP3β did not affect its binding to TDRD3 (Supplementary Fig. 10a). Therefore, Arg472 may function for higher neural activities by an unidentified molecular mechanism.

Decatenation of the DNA occurs in a relatively slow timescale. RMI1 promotes the TOP3α-catalyzed DNA decatenation, likely by retaining the open conformation of TOP3α^19^,^35^. The long insertion loop of RMI1 has been proposed to be important for stabilizing TOP3α in the open conformation to hold nicked DNAs^35^. Indeed, in the TOP3α–RMI1 structure, the long insertion loop is located in close proximity to the position corresponding to the catalytic decatenation loop, which is present exclusively in bacterial TOP3 and missing in eukaryotic TOP3^42^. It has been suggested that RMI1 could provide the decatenation element *in trans*^35^. In contrast to the decatenation, relaxation of the DNA occurs in a relatively fast timescale, and therefore is likely to be accompanied by the fast turnover of the open-closed conformations of the TOPO domain^19^. The TOP3β–TDRD3 complex functions only in relaxing mRNAs or resolving the transcription-associated R loop. TDRD3 does not slow down the DNA relaxation activity of TOP3β^32^ but rather enhances it^43^, in agreement with the short insertion loop of TDRD3, which is located far from the catalytic center composed of domains I, III and IV in the TOP3β–TDRD3 complex.

TOP3β recognizes both DNA and RNA as the substrates, whereas TOP3α recognizes only DNA^12^,^13^,^32^. However, the substrate-binding cavity of TOP3β is similar to that of TOP3α (Supplementary Fig. 2a–c). We could find no TOP3α- or TOP3β-specific structural element that could determine their substrate preferences. It seems unlikely that the difference in the substrate specificity between TOP3α and β is attributed to the difference in the architecture of their TOPO domains. Other regions of TOP3β or interacting factors might contribute to the dual specificity to DNA and RNA. Actually, the C-terminal RGG domain, which is unique to TOP3β, binds RNA with higher affinity than DNA and plays a critical role in the RNA topoisomerase activity of TOP3β^8^,^12^. A recent study also showed that TDRD3 could bind both DNA and RNA single strands^43^, although the DNA/RNA-binding region of TDRD3 has been unidentified. We found a positively charged area that could be considered as the potential DNA/RNA-binding site in DUF–OB of TDRD3 (Supplementary Fig. 10b). This region and/or a previously uncharacterized region of TDRD3 might interact with DNA and RNA. Additionally, the amino-acid sequence of the tandem ZF domain of TOP3β is substantially different from that of TOP3α, implying their functional difference. Although the functional role of the ZF domains has remained unclear for TOP3α or β, the DNA-bound structure of the *E. coli* TOP1 TOPO and ZF domains has shown the involvement of the ZF domain in the catalysis^44^. Further functional and structural studies of the ZF and RGG domains of TOP3β and the uncharacterized region of TDRD3 may be required for better understanding of the dual specificity of TOP3β to RNA and DNA.

## Methods

### Protein preparation

The cDNAs encoding human TOP3β TOPO domain (1–612), human TDRD3 OB-fold domain (48–171), DUF and OB-fold domains (1–161, 1–171, or 1–180), and human RMI1 DUF and OB-fold domains (1–219) were PCR-amplified from a human cDNA library (Human Brain, whole QUICK-Clone cDNA, Clontech). The amplified TOP3β cDNA was cloned into pFastBac Dual expression vector (Invitrogen) with the N-terminal GST tag. The amplified TDRD3 and RMI1 cDNAs were cloned into pCold I expression vector (Takara) with the N-terminal GST and His_6_-SUMO tags, respectively.

The GST-tagged TOP3β was expressed in baculovirus-infected Sf9 cells, following the manufacturer’s instruction for the Bac-to-Bac system (Invitrogen), except that X-treme GENE HP (Roch) was used as the transfection reagent for production of P1 virus. The harvested cells were lysed by sonication in 25 mM Tris-Cl buffer (pH 7.4) containing 300 mM NaCl and 5 mM β-mercaptoethanol. The cleared lysate was loaded onto a Glutathione Sepharose 4 FF (GE Healthcare) column pre-equilibrated with 25 mM Tris-Cl buffer (pH 7.4) containing 300 mM NaCl, 1 mM MgCl_2_ and 5 mM β-mercaptoethanol. The column was washed with the same buffer. The bound protein was eluted with 25 mM Tris-Cl buffer (pH 7.4) containing 300 mM NaCl, 1 mM MgCl_2_, 5 mM β-mercaptoethanol and 15 mM reduced glutathione. The GST tag of the eluted protein was removed by HRV 3 C protease. The TOP3β protein was further purified by a size-exclusion chromatography on a HiLoad 16/60 Superdex 200 prep grade (GE Healthcare) column equilibrated with 25 mM Tris-Cl buffer (pH 7.4) containing 200 mM NaCl, 1 mM MgCl_2_ and 5 mM β-mercaptoethanol. The purified TOPO domain of TOP3β was concentrated to 2–8 g/L for crystallization.

The GST-fused DUF–OB of TDRD3 was expressed in *E. coli* Rosetta (DE3) strain (invitrogen) and purified in the same manner as TOP3β, followed by an additional purification step using a Glutathione Sepharose FF column to remove the remaining GST. The purified DUF–OB of TDRD3 was concentrated to 3 g/L for crystallization.

The His_6_-SUMO-tagged DUF–OB of RMI1 was expressed in *E. coli* Rosetta (DE3) strain. The cell was lysed by sonication with 25 mM Tris-Cl buffer (pH 7.4) containing 400 mM NaCl, 2 mM MgCl_2_, 5 mM imidazole, 5 mM β-mercaptoethanol and 0.5% Triton X-100. The cleared lysate was loaded onto a Ni-NTA Superflow (Qiagen) column pre-equilibrated with the sonication buffer without Triton X-100. The column was washed with 25 mM Tris-Cl buffer (pH 7.4) containing 200 mM NaCl, 2 mM MgCl_2_, 50 mM imidazole and 5 mM β-mercaptoethanol. The bound protein was eluted with 25 mM Tris-Cl buffer (pH 7.4) containing 200 mM NaCl, 2 mM MgCl_2_, 250 mM imidazole and 5 mM β-mercaptoethanol. The His_6_-SUMO tag of the eluted protein was removed by Ulp1 protease. The RMI1 protein was dialyzed against 25 mM Tris-Cl buffer (pH 7.4) containing 200 mM NaCl, 2 mM MgCl_2_ and 5 mM β-mercaptoethanol, and loaded again onto a Ni-NTA Superflow (Qiagen) column to remove the remaining His_6_-SUMO tag.

The TOP3β–TDRD3 complex was prepared by mixing TOP3β (1–612) and an excess amount of TDRD3 (1–171). The complex was treated with 0.2% (w/w, protease/sample) chymotrypsin at 20 °C for 1 hr to trim the C-terminal unstructured region of the TDRD3 DUF domain, and was purified by size-exclusion chromatography on a HiLoad 16/60 Superdex 200 prep grade (GE Healthcare) column equilibrated with 25 mM Tris-Cl buffer (pH 7.4) containing 200 mM NaCl, 1 mM MgCl_2_ and 5 mM β-mercaptoethanol. The purified TOP3β–TDRD3 complex was concentrated to 8 g/L for crystallization.

### Crystallization

All samples were crystallized by the sitting drop vapor diffusion method at 4 °C (TOP3β TOPO) or 20 °C (TDRD3 DUF–OB and the TOP3β–TDRD3 complex). Protein solutions were mixed with equal volumes of the following reservoir solutions: 0.1 M HEPES-Na (pH 7.5), 0.2 M MgCl_2_ and 25% PEG3350 for the TOP3β TOPO domain; 1.26 M NaH_2_PO_4_ and 0.14 M K_2_HPO_4_ (pH 5.6) for the TDRD3 DUF–OB domains; 50 mM Na-cacodylate (pH 6.2), 10 mM MgSO_4_ and 1.7 M Li_2_SO_4_ for the TOP3β–TDRD3 complex. The crystals of the TOP3β TOPO domain, the TDRD3 DUF–OB domains or the TOP3β–TDRD3 complex were cryoprotected by supplementation of the reservoir solutions with the final concentrations of 20% PEG400, 30% ethylene glycol or 25% ethylene glycol, respectively, before flash cooling in liquid N_2_.

### Data collection and structure determination

All diffraction data sets were collected at 100 K at beamline BL41XU of SPring-8 (Hyogo, Japan) and were processed with HKL2000^45^ and CCP4 program suite. Data collection and refinement statistics are shown in Supplementary Table 1. All structures were solved by the molecular replacement method using Molrep^46^. Human TOP3α structure from the TOP3α–RMI1 complex (PDBID: 4CGY)^35^ was used as the search model for structure determination of the TOP3β TOPO domain. The structures of human RMI1 DUF–OB (PDBID: 3NBI)^47^ and the TOP3β TOPO domain were used as the search models for the initial structure determination of the TOP3β–TDRD3 complex from a preliminary 4.5-Å resolution data set. At this stage, the relative orientation between the DUF1767 and OB-fold domains of TDRD3 could be refined by rigid-body refinement by Phenix software^48^. This refined DUF–OB structure of TDRD3 was used as the search model for high-resolution structure determination of TDRD3 DUF–OB. The final refined TOP3β TOPO and TDRD3 DUF–OB structures were used as the search models for structure determination of the TOP3β–TDRD3 complex. Model building and autocorrection/refinement were carried out using the programs Coot^49^ and Phenix, respectively. The coordinates of TOP3β (1–612), TDRD3 (1–161) and the TOP3β (1–612)–TDRD3 (1–161) complex were deposited in the Protein Data Bank with the accession numbers 5GVC, 5GVD, and 5GVE, respectively.

### Limited proteolytic analysis

0.3 g/L samples (TDRD3 DUF–OB [1–161, 1–171 or 1–180] or their complexes with TOP3β [1–612]) were incubated with 0.2% (w/w, protease/sample) chymotrypsin at 20 °C. Two aliquots of the samples were taken after 0.5 and 2 hrs for SDS-PAGE analysis. The reactions were stopped by addition of SDS-PAGE sample loading buffers containing 4 mM EDTA.

### Pull-down assay

Glutathione Sepharose FF (GE healthcare) resins with bound wild-type GST-TOP3β (1–612) were incubated with the wild-type or mutant TDRD3 DUF–OB (1–161 or 1–171) in 20 mM Tris-Cl (pH 7.4) buffer containing 200 mM NaCl, 1 mM MgCl_2_, 5 mM β-mercaptoethanol and 0.1% Triton X-100 at 4 °C for 2 hrs. The resins were washed with the same buffer four times. The TDRD3 bound to the resins was co-eluted with GST-TOP3β (1–612) with 50 mM Tris-Cl (pH 8.0) buffer containing 200 mM NaCl, 1 mM MgCl_2_, 5 mM β-mercaptoethanol and 15 mM reduced glutathione. The eluted TDRD3 was detected by SDS-PAGE with Coomassie brilliant blue staining. The gels were imaged in 8-bit TIFF format. The pixel densities of the gel bands of the bound proteins were analyzed by the ImageJ software^50^. The experiments were repeated three times for quantification of the bands of the bound proteins.

## Additional Information

**How to cite this article:** Goto-Ito, S. *et al*. Structural basis of the interaction between Topoisomerase IIIβ and the TDRD3 auxiliary factor. *Sci. Rep.* **7**, 42123; doi: 10.1038/srep42123 (2017).

**Publisher's note:** Springer Nature remains neutral with regard to jurisdictional claims in published maps and institutional affiliations.

## Supplementary Material

Supplementary Table and Figures
