# Supplementary material for: Structural basis of the interaction between Topoisomerase IIIβ and the TDRD3 auxiliary factor
Source: Sci Rep. 2017 Feb 8;7:42123. doi: 10.1038/srep42123 (PMC5296760; doi:10.1038/srep42123)
Supplement: Supplementary Table and Figures [file srep42123-s1.pdf]

Supplementary Information

Structural basis of the interaction between Topoisomerase IIIβ and the TDRD3 auxiliary factor

Sakurako Goto-Ito[1,3], Atsushi Yamagata[1,2,3],Tomio S. Takashi[1,3], Yusuke Sato[1,2,3], Shuya Fukai[1,2,3,*]

[1]Structural Biology Laboratory, Structural Life Science Division, Synchrotron Radiation Research Organization and Institute of Molecular and Cellular Biosciences, The University of Tokyo, Tokyo 113-0032, Japan

[2]Department of Computational Biology and Medical Sciences, Graduate School of Frontier Sciences, The University of Tokyo, Chiba 277-8501, Japan

[3]CREST, JST, Saitama 332-0012, Japan

*Correspondence should be addressed to S.F. (fukai@iam.u-tokyo.ac.jp).

Supplementary Table 1. Data collection and refinement statistics.

Molecule name	TOP3β–TDRD3	TOP3β	TDRD3
PDB ID	5GVE	5GVC	5GVD
Data collection			
Beamline	SPring-8 BL41XU	SPring-8 BL41XU	SPring-8 BL41XU
Space group	$P6_5$	$C2$	$P6_5$
Cell constants			
a, b, c (Å)	173.6, 173.6, 111.8	176.1, 93.7, 91.6	85.7, 85.7, 104.2
α, β, γ (˚)	90, 90, 120	90, 91.9, 90	90, 90, 120
Resolution	50–3.6 (3.66–3.6)	50–2.44 (2.48–2.44)	50–1.62 (1.65–1.62)
R_{sym}	0.122 (0.681)	0.124 (0.439)	0.087 (0.872)
$I/\sigma I$	11.2 (1.56)	13.4 (3.89)	43.9 (2.86)
Redundancy	9.0 (8.0)	6.7 (6.3)	20.4 (20.0)
Completeness (%)	99.9 (99.9)	99.1 (98.3)	100 (100)
**Refinement**			
Resolution (Å)	50–3.6	50–2.44	50–1.62
No. reflections	22224	55090	54685
R_{work}/R_{free}	0.198/0.245	0.182/0.229	0.175/0.199
No. atoms			
Protein	6082	9591	2351
Ligand/ion	1	2	23
Water	-	411	241
B-factors (Å^2)			
Protein	153.2	39.8	29.4
Ligand/ion	114.4	30.2	30.6
Water	-	36.3	35.0
R.m.s.deviations			
Bond lengths (Å)	0.004	0.002	0.018
Bond angles (˚)	1.13	0.69	1.61

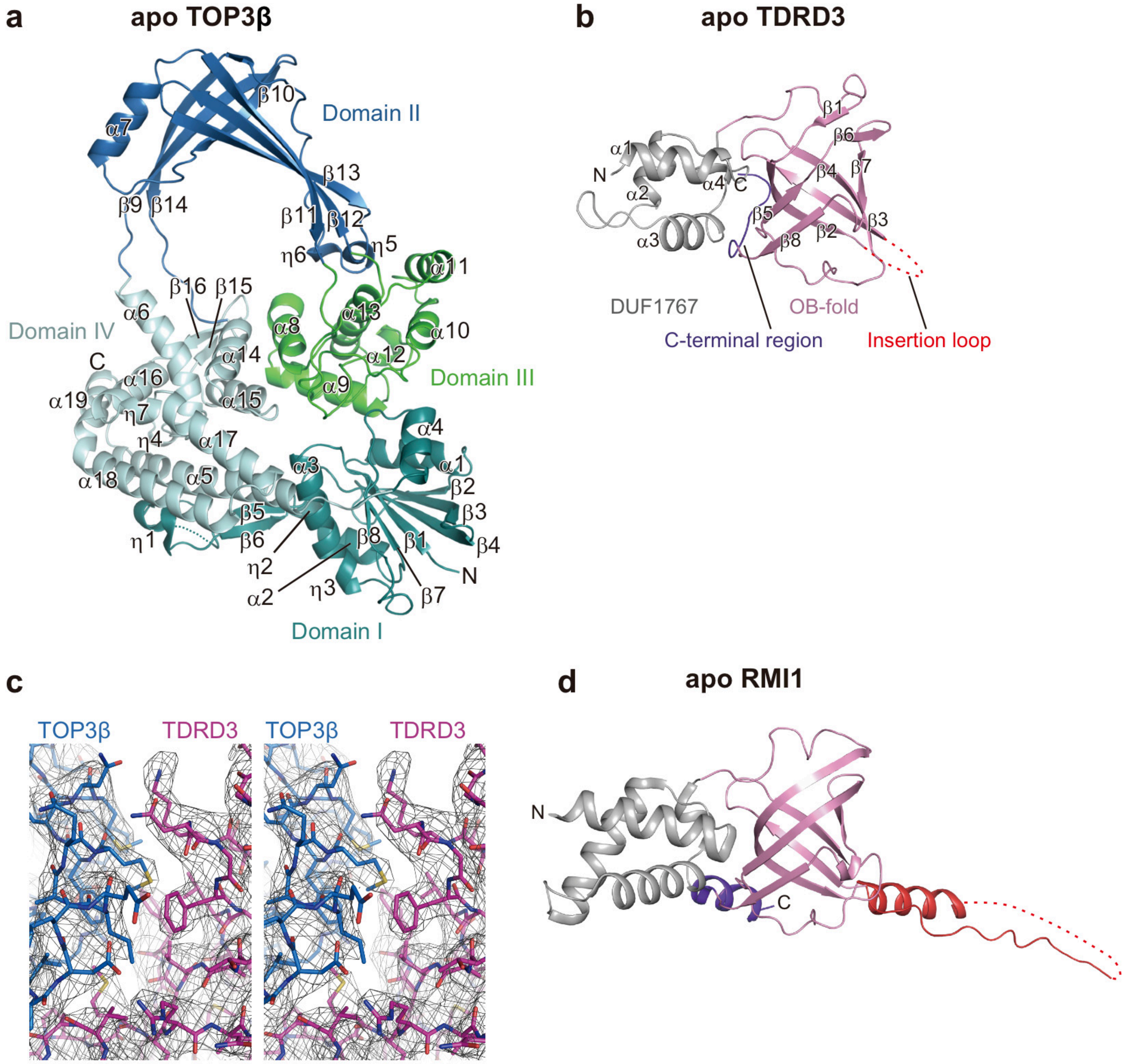

Supplementary Figure 1. Crystal structures of apo TOP3β and TDRD3.

(a) Crystal structure of the TOP3β TOPO domain.

(b) Crystal structure of the TDRD3 DUF–OB domains.

(c) $2F_O$-F_C electron density map around the TOP3β–TDRD3 interface, contoured at 1.1 σ level (stereo view).

TOP3β and TDRD3 are colored in blue and magenta, respectively.

(d) Crystal structure of the RMI1 DUF–OB domains.

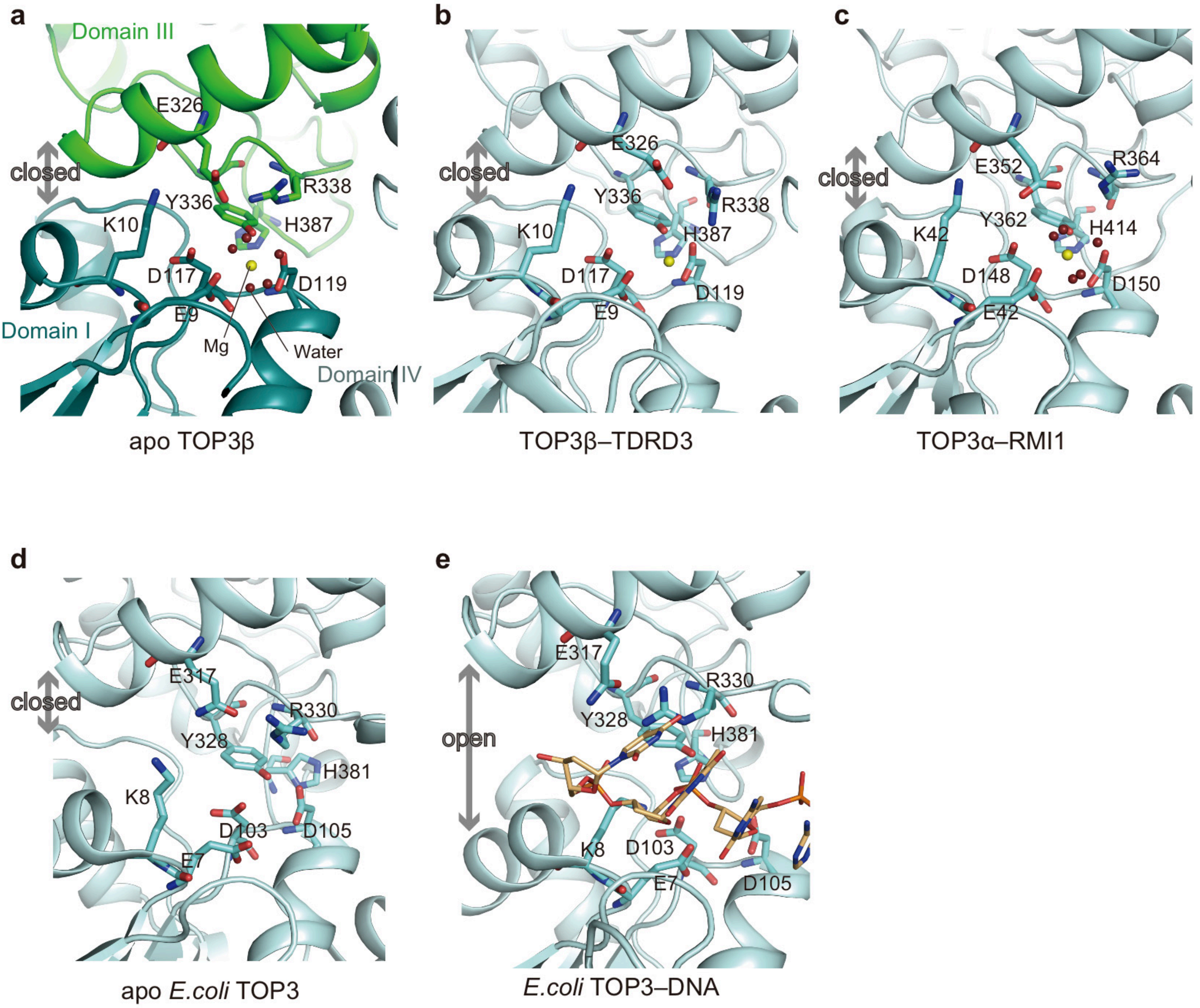

Supplementary Figure 2. Close-up views of the catalytic pockets in TOP3 structures.

(a) Catalytic pocket of apo TOP3β in the closed conformation. The catalytic residues are shown as sticks. The bound magnesium ion and its coordinated water are shown as yellow and red balls, respectively.

(b) Catalytic pocket of the TDRD3-bound TOP3β in the closed conformation. The representation scheme is the same as that in (A).

(c) Catalytic pocket of the RMI1-bound TOP3β in the closed conformation. The representation scheme is the same as that in (A).

(d) Catalytic pocket of apo bacterial TOP3 in the closed conformation. The catalytic residues are shown as sticks.

(e) Catalytic pocket of the DNA-bound bacterial TOP3 in the open conformation. The catalytic residues and bound DNA are shown as cyan and orange sticks, respectively.

a

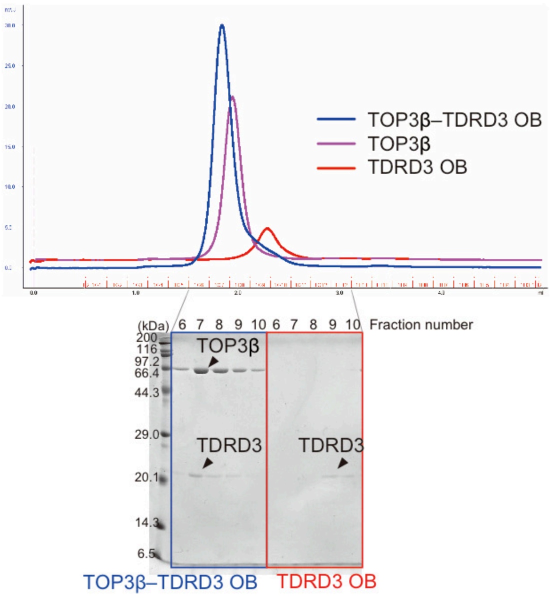

b

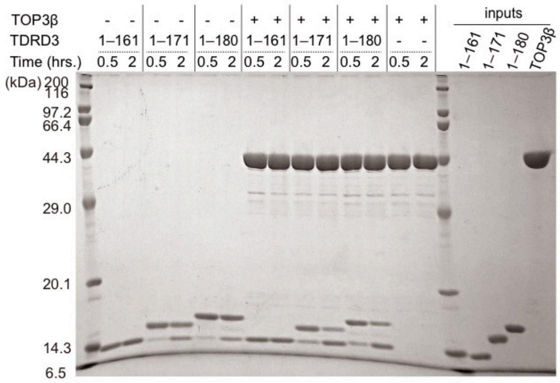

Supplementary Figure 3. Structural characterization of TDRD3.

(a) Elution profiles of the size-exclusion chromatography of the TOP3β TOPO domain (purple), the TDRD3 OB-fold domain (red) and their complex (blue). The peak fractions of TDRD3 OB-fold and the TOP3β TOPO–TDRD3 OB-fold complex were analyzed by SDS-PAGE.

(b) Limited chymotryptic digestion of TDRD3 DUF–OB (1–161, 1–171 or 1–180) in the presence or absence of TOP3β. Digestions of TDRD3 (1–171) and (1–180) produced the bands corresponding to TDRD3 (1–161), whereas TDRD3 (1–161) was not cleaved in the present condition.

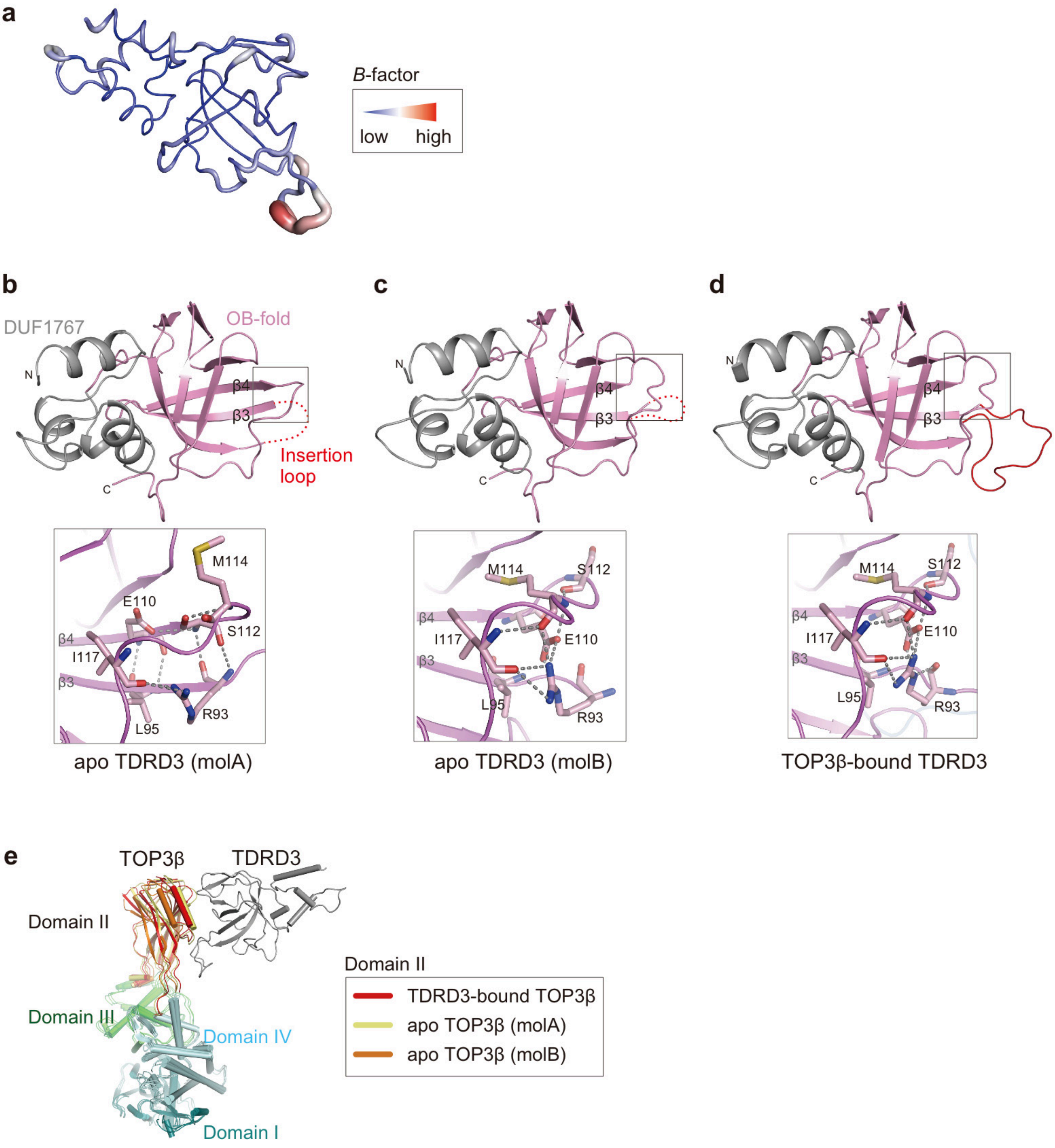

Supplementary Figure 4. Conformational changes in TDRD3 and TOP3β.

(a) *B*-factor putty representation of TDRD3 DUF–OB.

(b) Structure of apo TDRD3 DUF–OB (mol A). The close-up view shows hydrogen bonds (dotted lines) around the N- and C-terminal edges of β3 and β4, respectively. The residues involved in the hydrogen bonds are shown as sticks.

(c) Structure of apo TDRD3 DUF–OB (mol B). The representation scheme is the same as that in (b).

(d) Structure of the TOP3β-bound TDRD3 DUF–OB. The representation scheme is the same as that in (b).

(e) Structural comparison between the apo and TDRD3-bound TOP3β structures. Domains I, III and IV are colored in blue, cyan and green, respectively. Domains II of apo TOP3β (molA), apo TOP3β (molB) and the TDRD3-bound TOP3β are colored in red, yellow and orange, respectively. The bound TDRD3 is colored in grey.

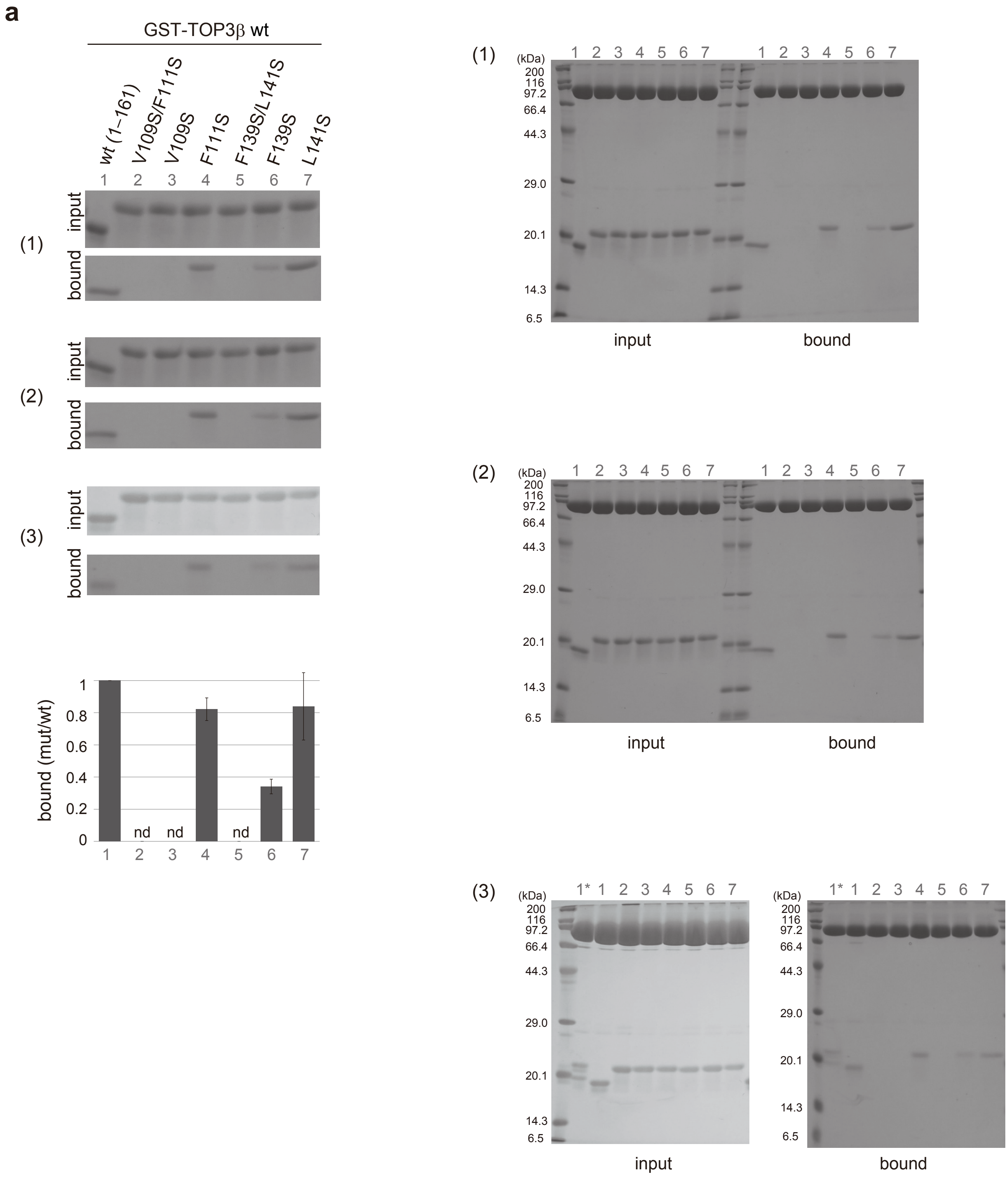

Supplementary Figure 5.

b

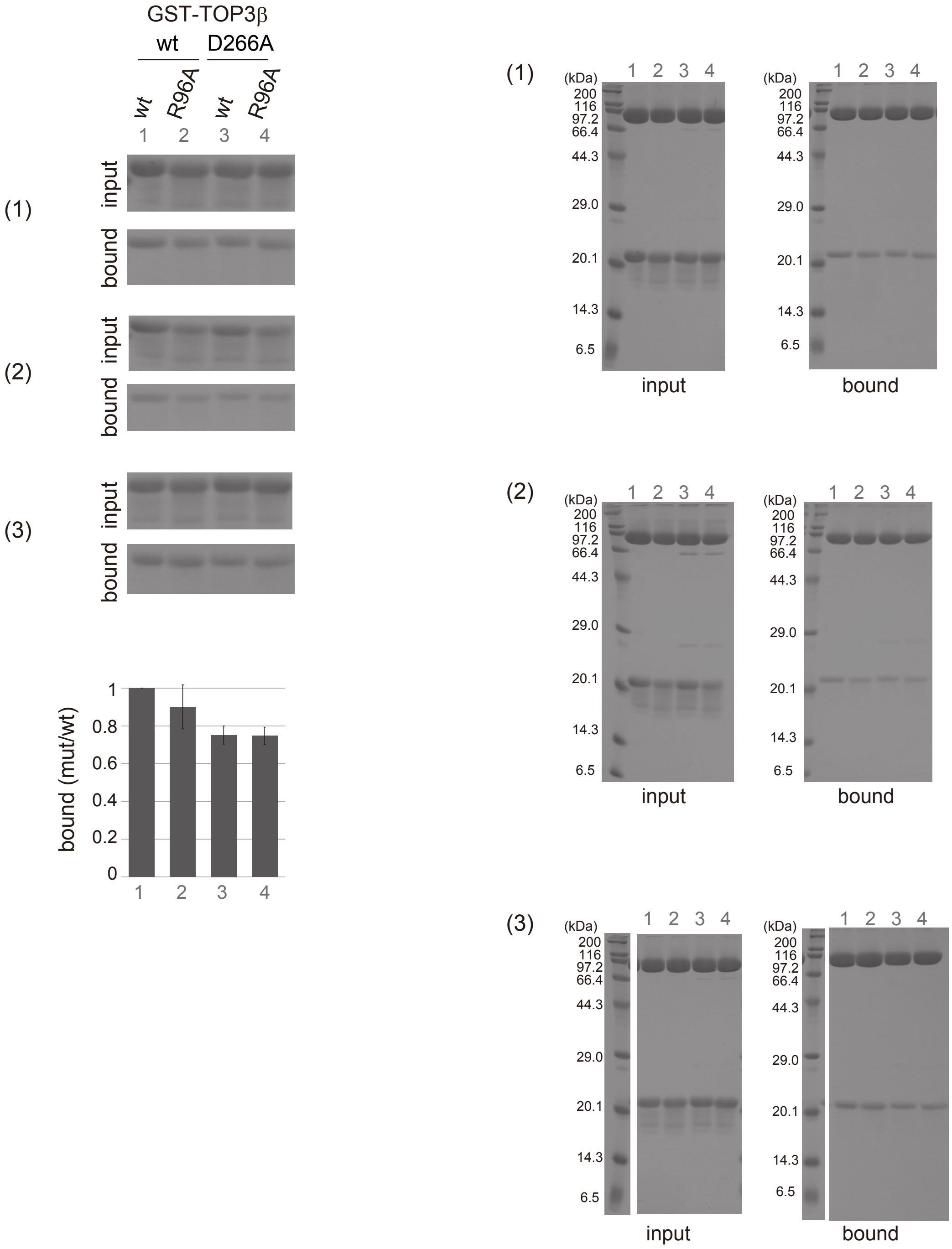

Supplementary Figure 5.

c

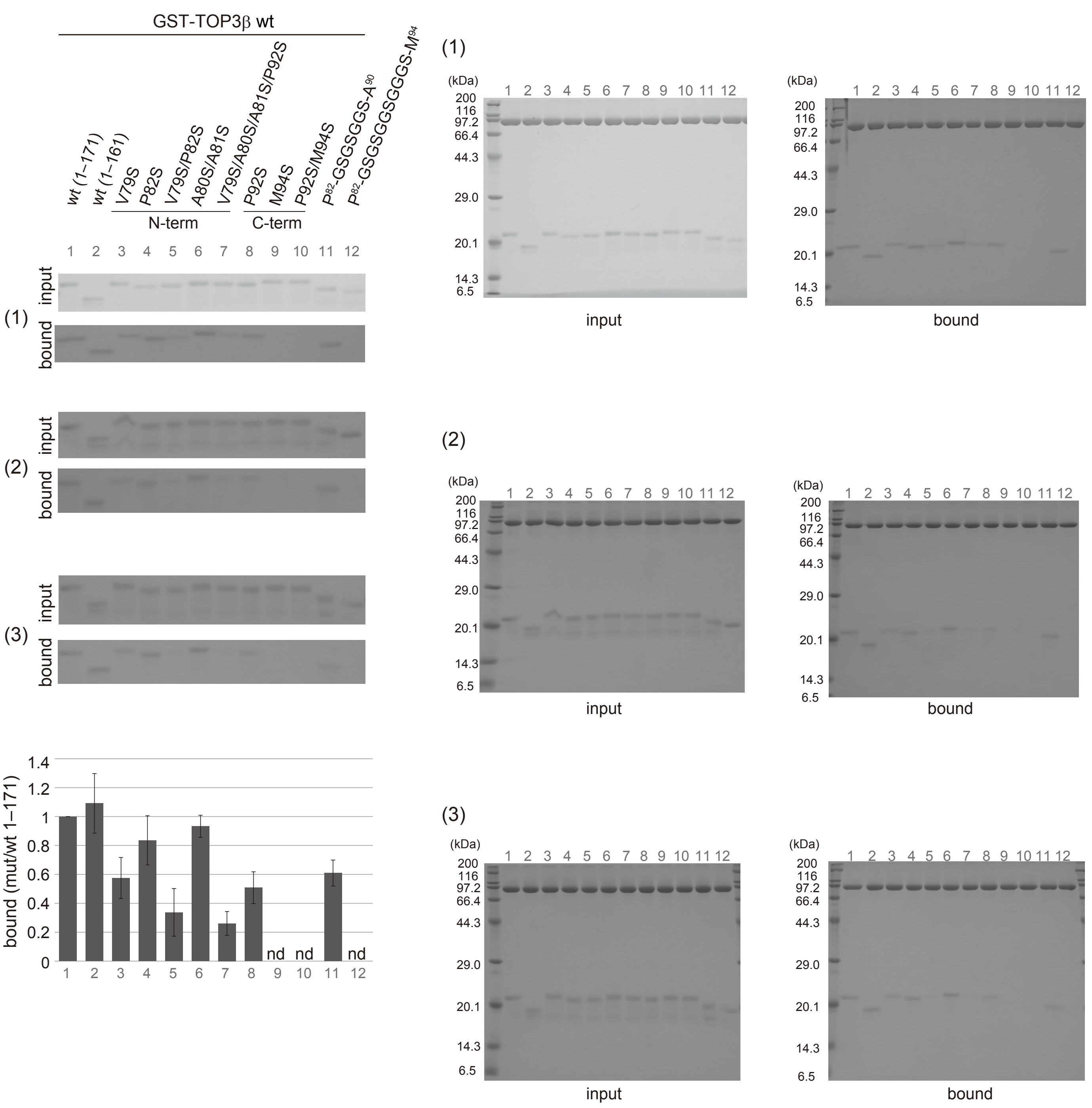

Supplementary Figure 5.

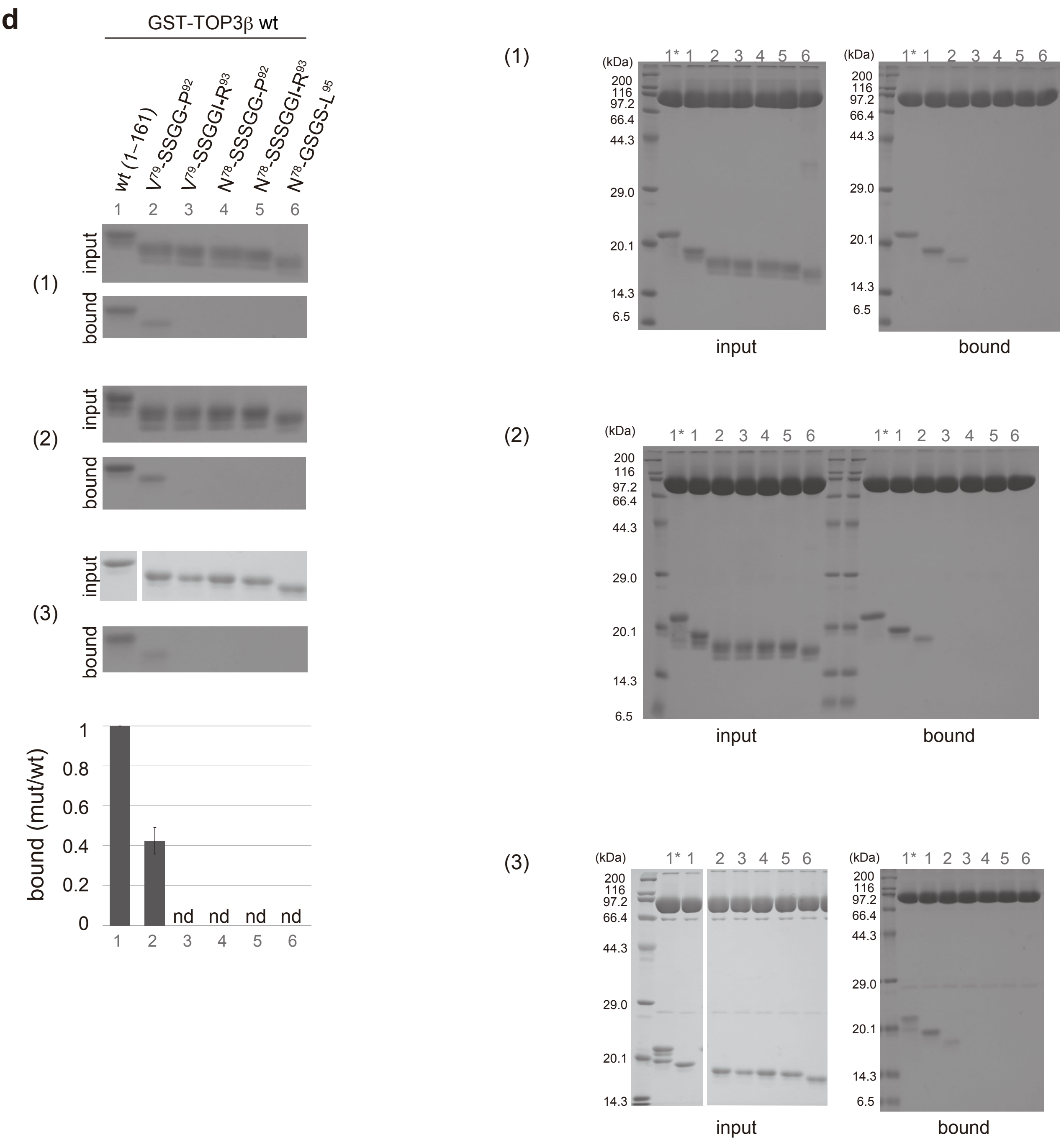

Supplementary Figure 5.

Quantitative analyses and original full-length gel images of GST-pulldown assays to assess the interaction of TDRD3 mutants with TOP3β.

The band intensities of the bound mutant proteins relative to the bound wild-type proteins from three independent experiments were measured by densitometry using the ImageJ software. The averaged relative intensities are presented as bar graphs (nd; not detectable). Error bars represent the standard deviations. Lanes 1* correspond to the experiments using wild-type TDRD3 (1–171) as inputs, which are not presented in Fig. 3.

(a) Quantitative analyses and original full-length gel images corresponding to the top panel of Fig. 3c.

(b) Quantitative analyses and original full-length gel images corresponding to the bottom panel of Fig. 3c.

(c) Quantitative analyses and original full-length gel images corresponding to the left panel of Fig. 3f.

(d) Quantitative analyses and original full-length gel images corresponding to the right panel of Fig. 3f.

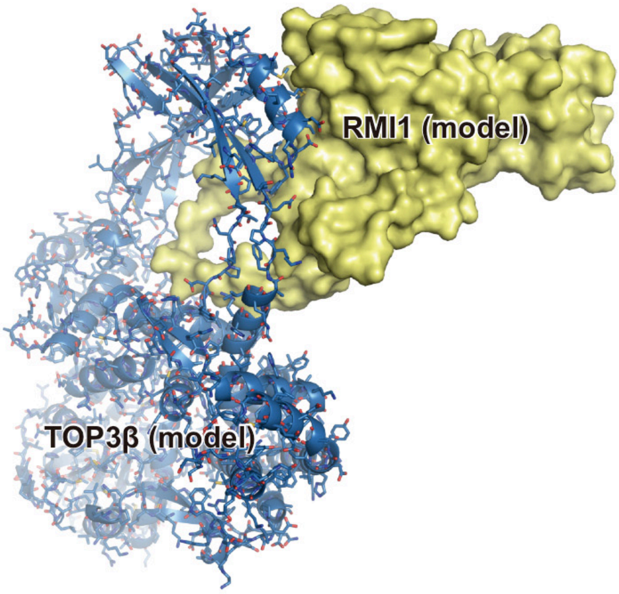

Supplementary Figure 6. Docking analysis between RMI1 and TOP3β.

The TOP3α–RMI1 complex was superposed onto the TOP3β–TDRD3 complex so as to minimize the Cα r.m.s.d. between TOP3α and β. TOP3β is shown as blue cartoon and stick models. RMI1 is shown as a yellow surface model.

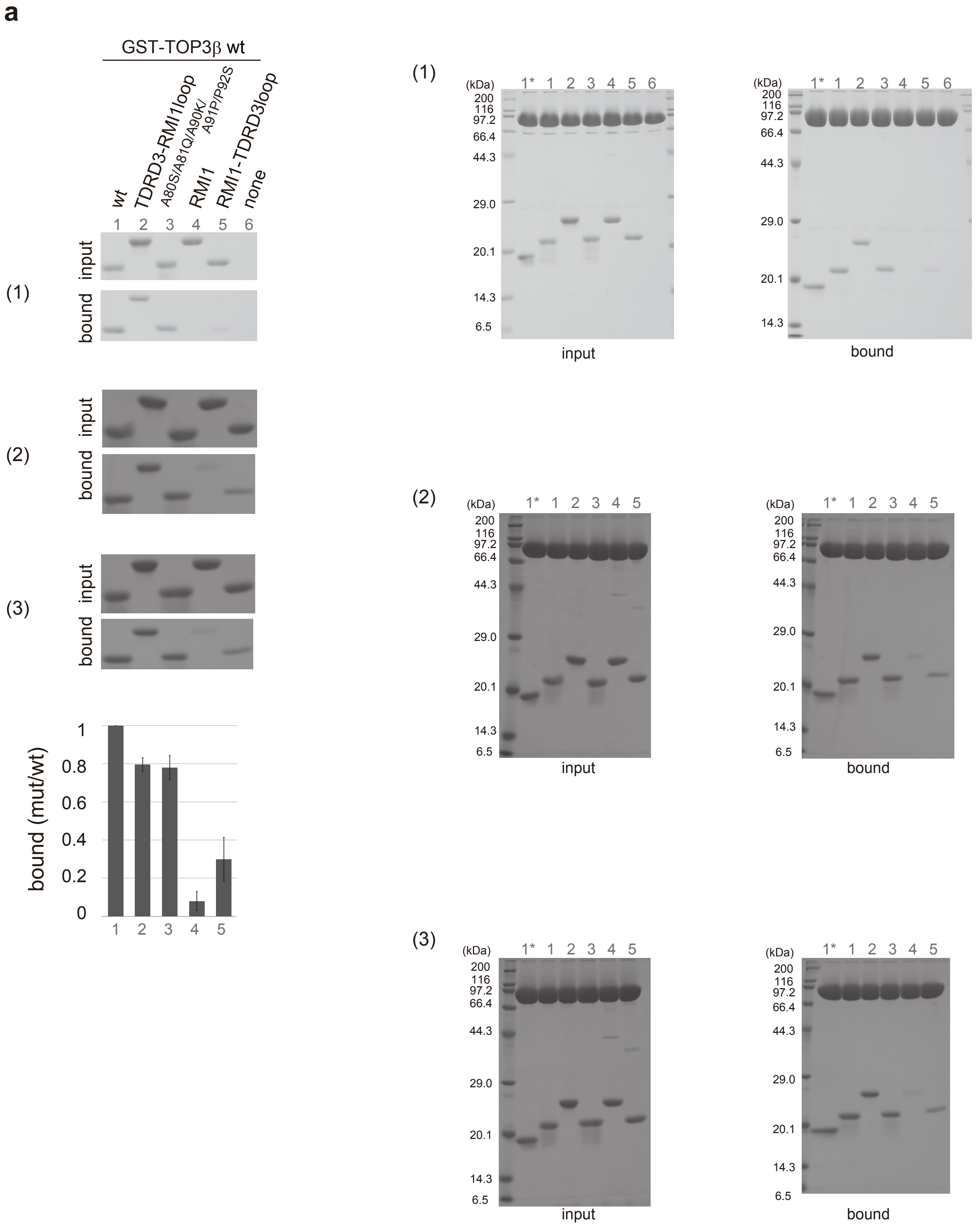

Supplementary Figure 7.

b

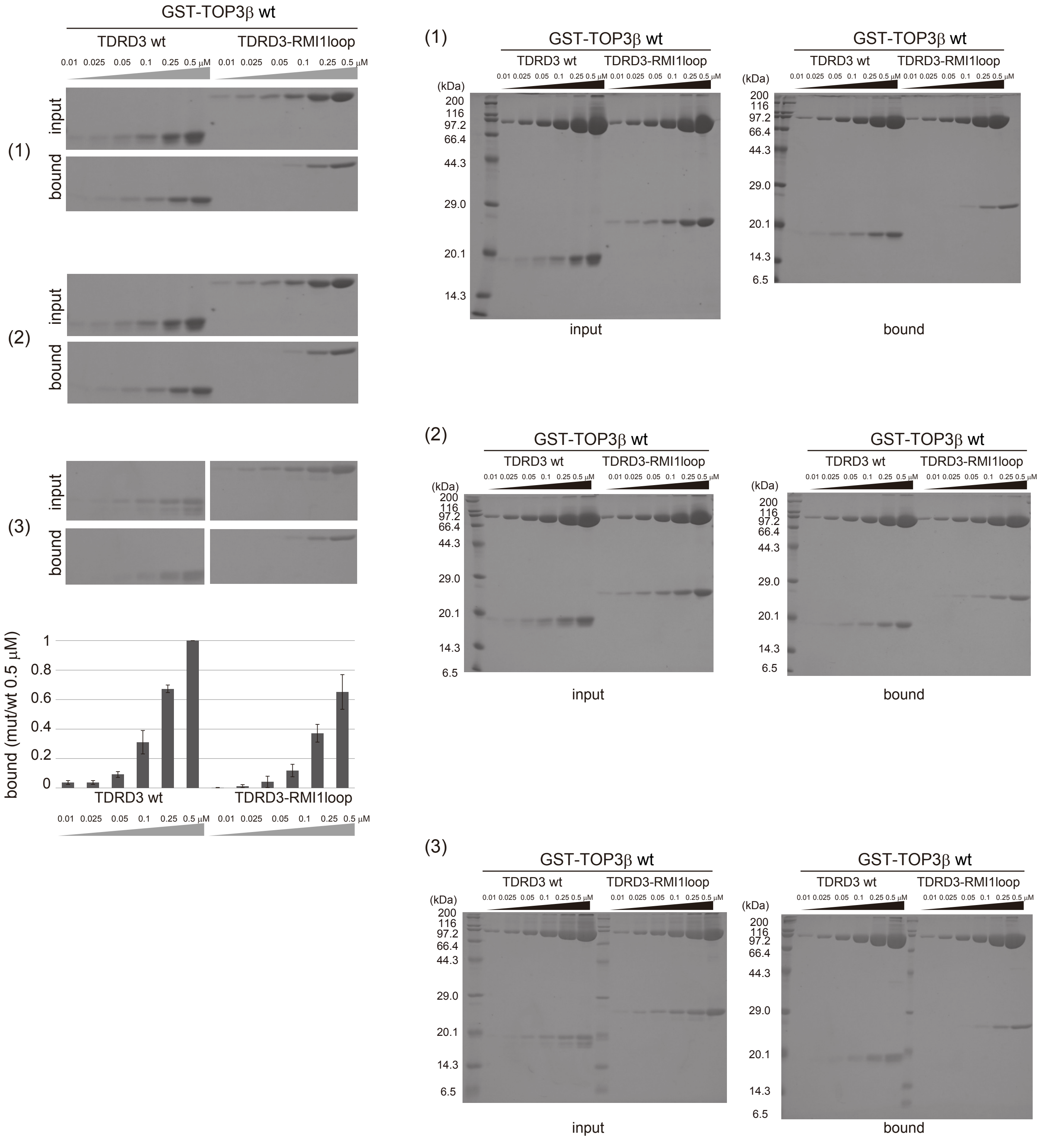

Supplementary Figure 7.

c

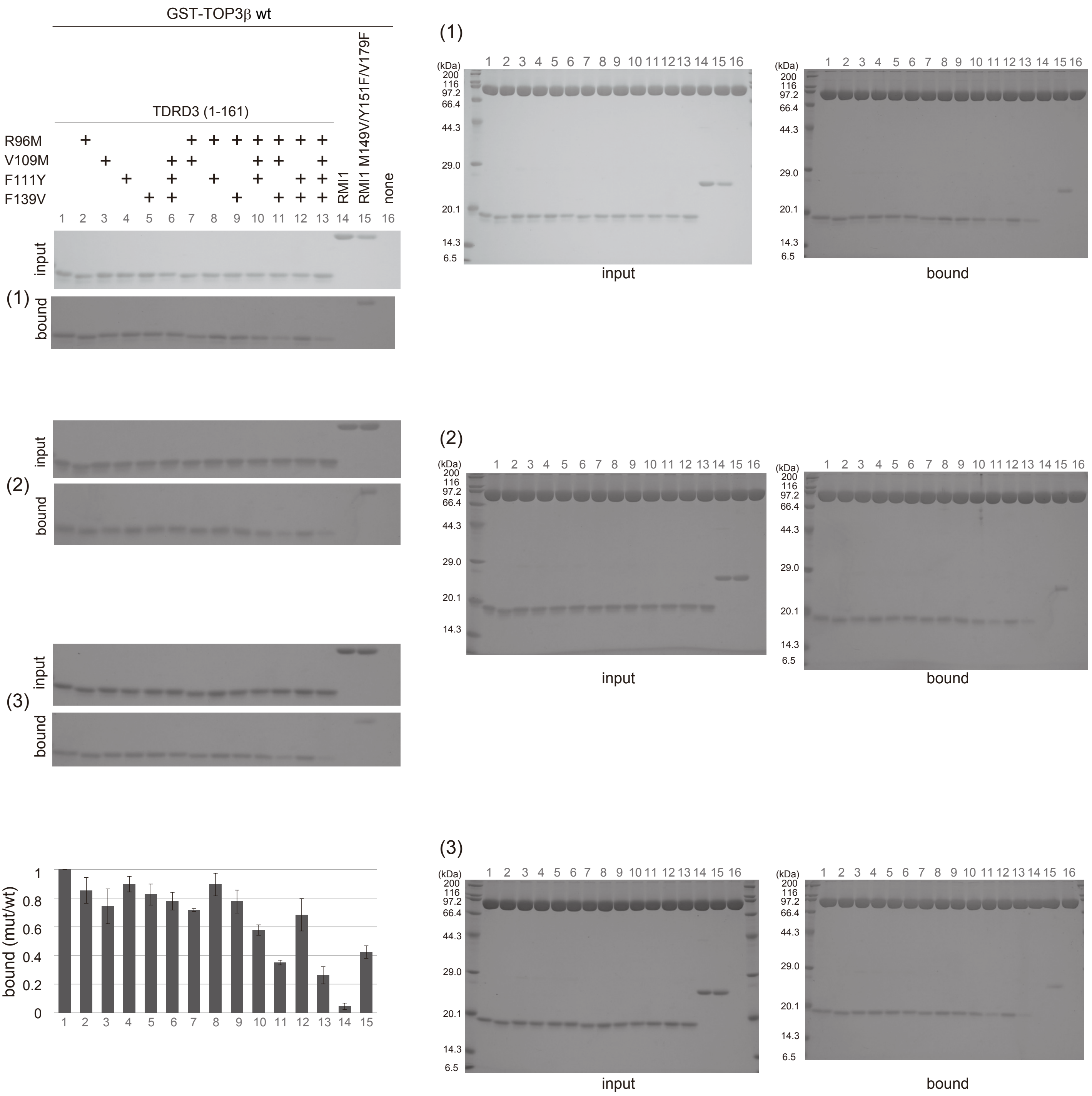

Supplementary Figure 7.

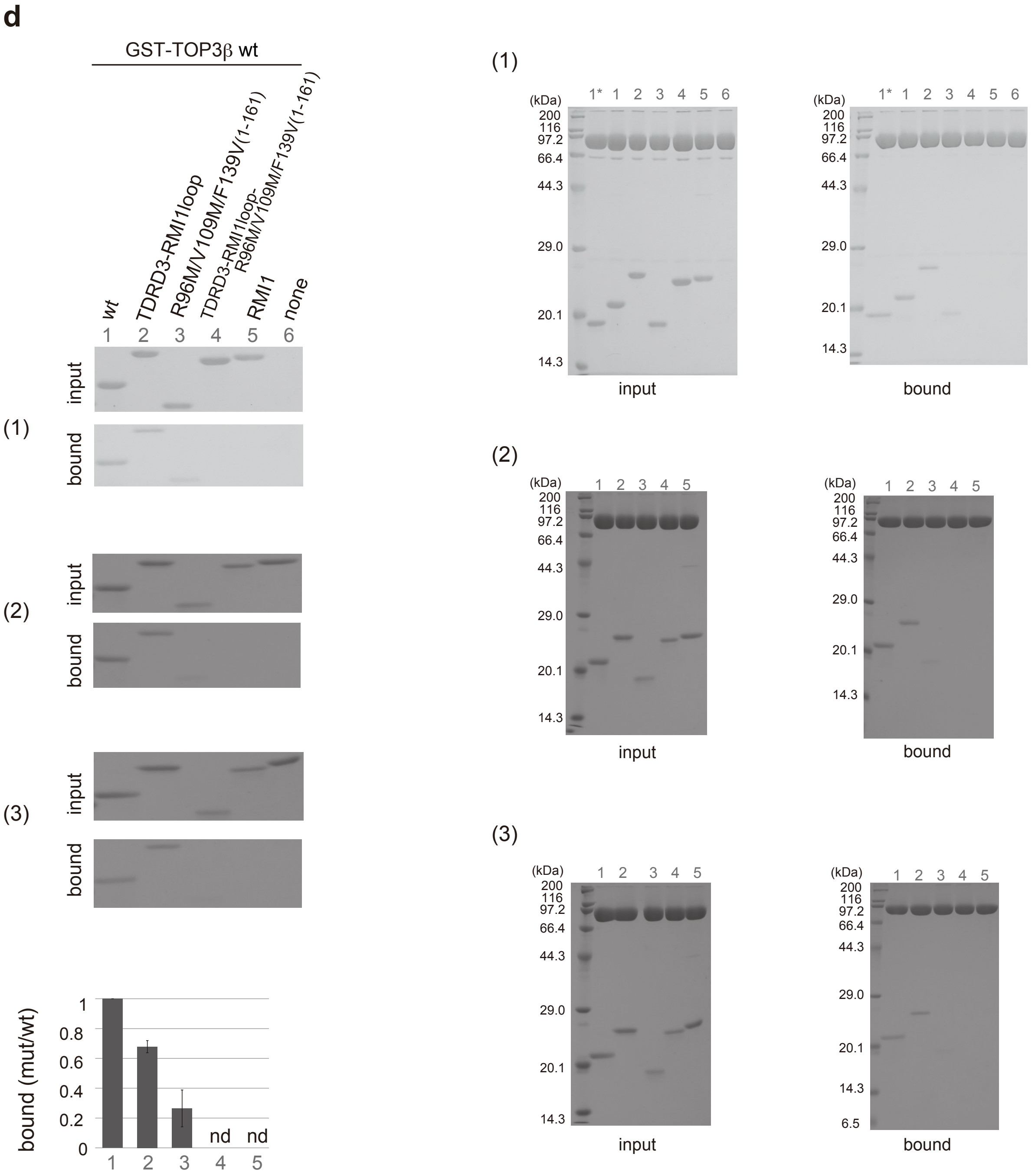

Supplementary Figure 7.

Quantitative analyses and original full-length gel images of GST-pull down assays to identify the specificity determinants for the interaction of TDRD3 with TOP3β.

The band intensities of the bound mutant proteins relative to the bound wild-type proteins from three independent experiments were measured by densitometry using the ImageJ software. The averaged relative intensities are presented as bar graphs (nd; not detectable). Error bars represent the standard deviations. Lanes 1* correspond to the experiments using wild-type TDRD3 (1–161) as inputs, which are not presented in Fig. 4.

(a) Quantitative analyses and original full-length gel images corresponding to Fig. 4b.

(b) Quantitative analyses and original full-length gel images corresponding to Fig. 4c.

(c) Quantitative analyses and original full-length gel images corresponding to Fig. 4d.

(d) Quantitative analyses and original full-length gel images corresponding to Fig. 4e.

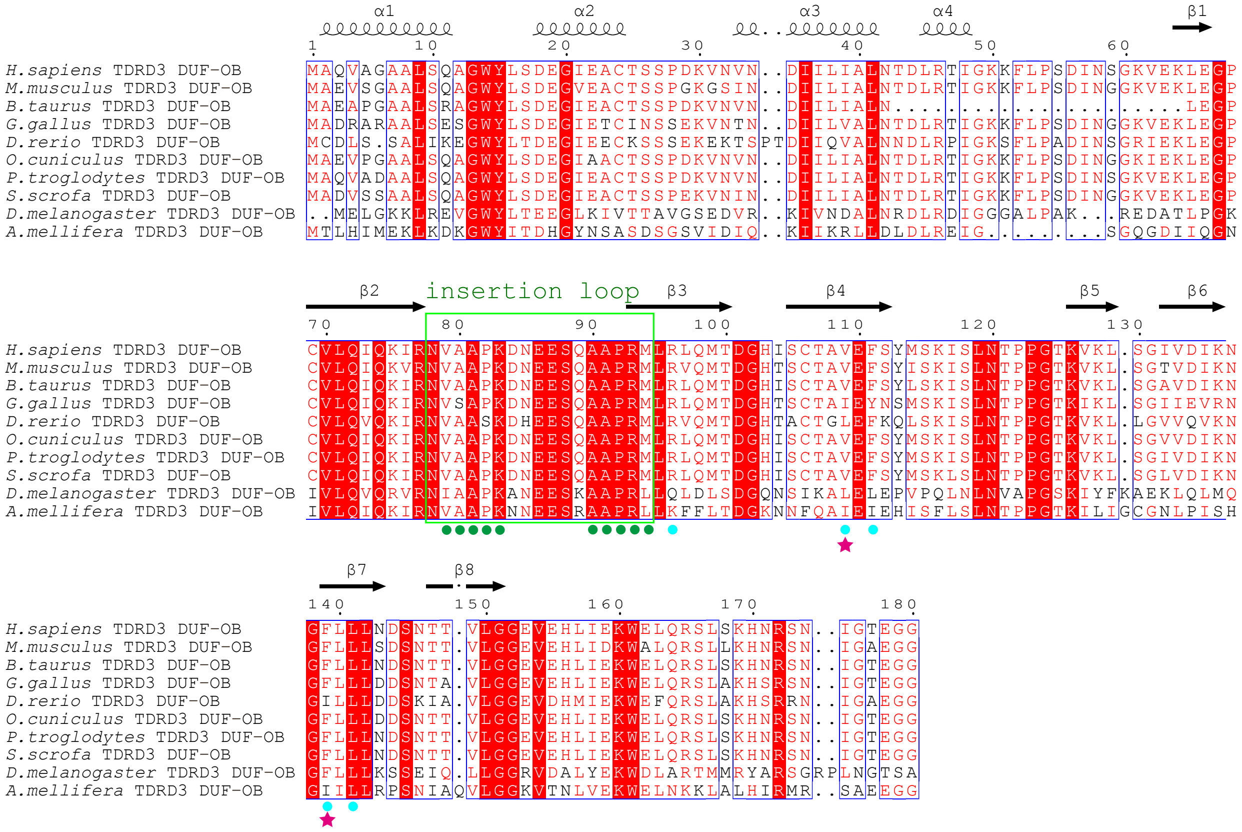

Supplementary Figure 8. Amino-acid sequence alignment of the TDRD3 DUF–OB domains.

The TOP3β-interacting residues in the insertion loop and core region are marked with green and cyan dots, respectively. The residues responsible for the preference towards TOP3β are marked with magenta stars. 100% and more than 70% identical residues are highlighted with red backgrounds and red characters, respectively. The secondary structure and residue numbers of human TDRD3 are shown above the alignment.

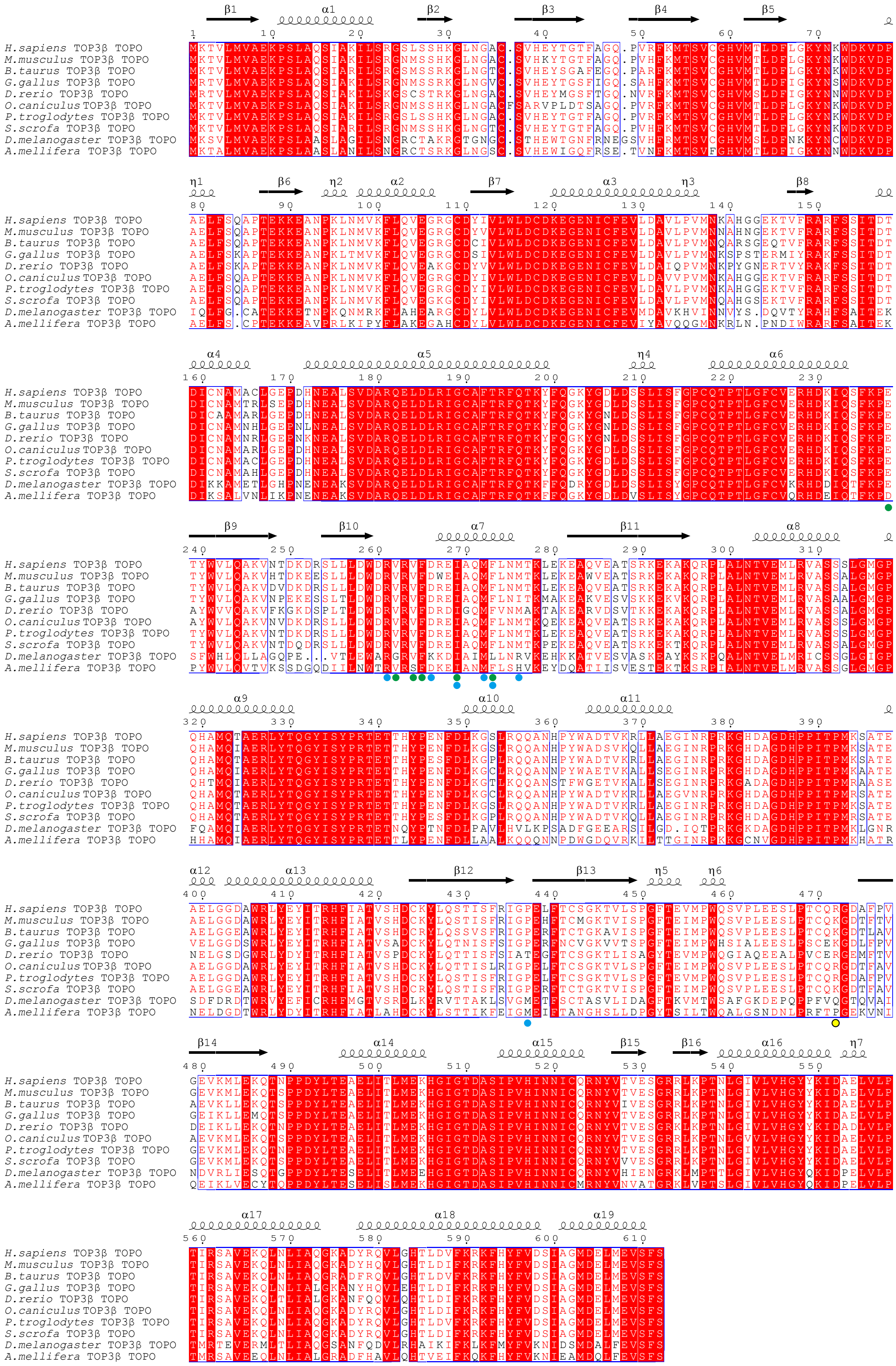

Supplementary Figure 9. Amino-acid sequence alignment of the TOP3β TOPO domain.

The residues interacting with the insertion loop and core region of TDRD3 are marked in green and cyan dots, respectively. The residue whose mutation found in schizophrenia patients is marked with a yellow dot. 100% and more than 70% identical residues are highlighted with red backgrounds and red characters, respectively. The secondary structure and residue numbers of human TOP3β are shown above the alignment.

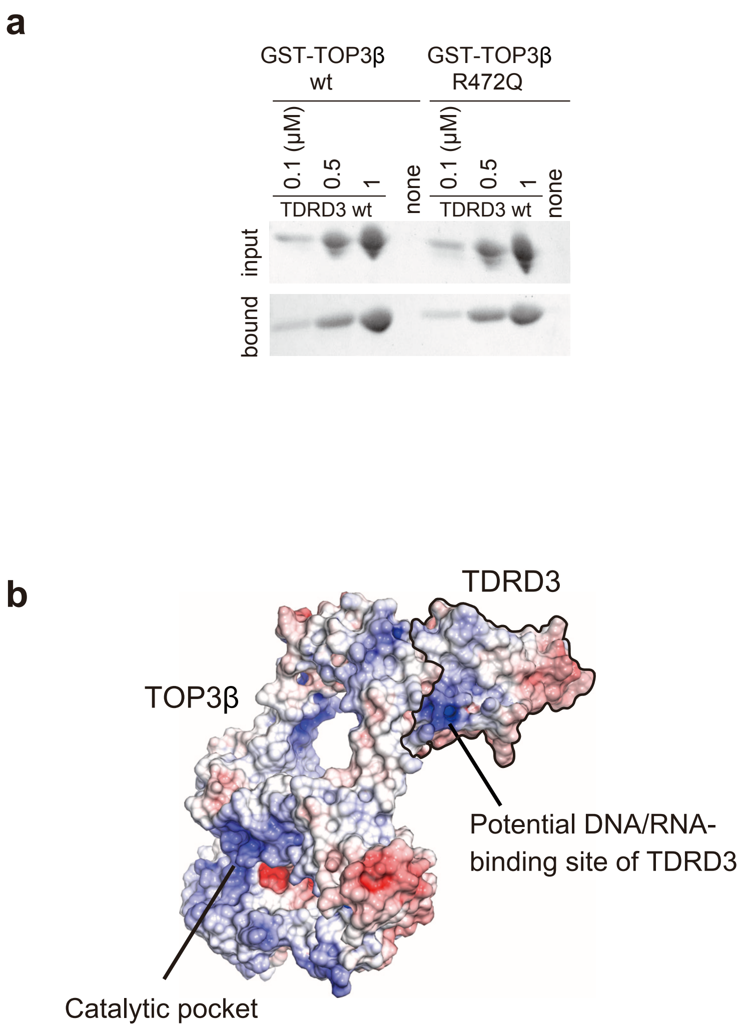

Supplementary Figure 10.

Schizophrenia-related mutation of TOP3β and a putative DNA/RNA-binding site of TDRD3

(a) GST pull-down assay of a TOP3β mutant containing a schizophrenia-related mutation. The GST-TOP3β-bound resin (wild type or R472Q) was incubated with 0.1, 0.5 or 1.0 μM TDRD3. The R472Q mutation of TOP3β did not affect binding to TDRD3.

(b) Surface electrostatic potential of TOP3β-TDRD3. The positive and negative charges are shown in blue and red, respectively, contoured at +10 (blue) to −10 (red) $k_B T/e$.
